# FDW028, a novel FUT8 inhibitor, impels lysosomal proteolysis of B7-H3 *via* chaperone-mediated autophagy pathway and exhibits potent efficacy against metastatic colorectal cancer

**DOI:** 10.1038/s41419-023-06027-0

**Published:** 2023-08-03

**Authors:** Mengmeng Wang, Zhoudong Zhang, Mengxi Chen, Yixin Lv, Sheng Tian, Fanyi Meng, Yawen Zhang, Xuqin Guo, Yinshuang Chen, Man Yang, Jiawei Li, Tian Qiu, Fang Xu, Zhi Li, Qi Zhang, Jie Yang, Jing Sun, Hongjian Zhang, Haiyang Zhang, Huanqiu Li, Weipeng Wang

**Affiliations:** 1 College of Pharmaceutical Sciences, Soochow University, Suzhou, 215123 China; 2 Department of Pharmacy, The Second Affiliated Hospital of Soochow University, Soochow University, Suzhou, 215123 China; 3 Institute of Medical Technology, Suzhou Vocational Health College, Suzhou, 215009 China

**Keywords:** Drug development, Colorectal cancer, Medicinal chemistry, Glycosylation

## Abstract

Metastatic colorectal cancer (mCRC) is a major cause of cancer-related mortality due to the absence of effective therapeutics. Thus, it is urgent to discover new drugs for mCRC. Fucosyltransferase 8 (FUT8) is a potential therapeutic target with high level in most malignant cancers including CRC. FUT8 mediates the core fucosylation of CD276 (B7-H3), a key immune checkpoint molecule (ICM), in CRC. FUT8-silence-induced defucosylation at N104 on B7-H3 attracts heat shock protein family A member 8 (HSPA8, also known as HSC70) to bind with 106-110 SLRLQ motif and consequently propels lysosomal proteolysis of B7-H3 through the chaperone-mediated autophagy (CMA) pathway. Then we report the development and characterization of a potent and highly selective small-molecule inhibitor of FUT8, named FDW028, which evidently prolongs the survival of mice with CRC pulmonary metastases (CRPM). FDW028 exhibits potent anti-tumor activity by defucosylation and impelling lysosomal degradation of B7-H3 through the CMA pathway. Taken together, FUT8 inhibition destabilizes B7-H3 through CMA-mediated lysosomal proteolysis, and FDW028 acts as a potent therapeutic candidate against mCRC by targeting FUT8.

**Figure:**
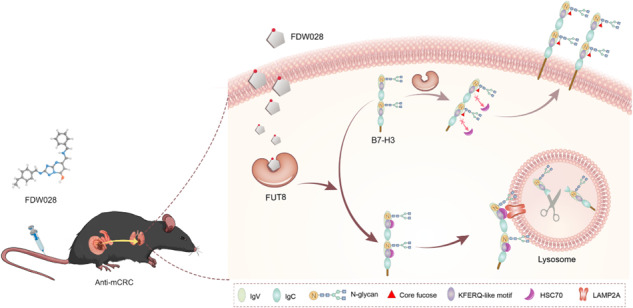
FDW028, an inhibitor specifically targeted FUT8, promotes defucosylation and consequent HSC70/LAMP2A-mediated lysosomal degradation of B7-H3, and exhibits potent anti-mCRC activities.

FDW028, an inhibitor specifically targeted FUT8, promotes defucosylation and consequent HSC70/LAMP2A-mediated lysosomal degradation of B7-H3, and exhibits potent anti-mCRC activities.

## Introduction

Nowadays the incidence and mortality of CRC rank the third and the second among cancers, respectively [1]. Approximately 50% of patients with initially localized CRC will develop metastases, which are incurable with a 5-year survival typically not exceeding 10% due to the lack of effective therapeutics [2]. Worse still, the incidence and mortality of CRC are rising [3], especially among young people [4]. Hence, it is urgent to discover new therapeutic strategies and develop effective drugs for the treatment of mCRC.

Glycosylation is thought to be one of the most prevalent posttranslational modification on proteins. Aberrant glycosylation is a feature of cancer cells and crucial in the pathological processes of malignant transformation, metastasis and immune evasion [5]. One prominent aberrant glycosylaion identified in malignancies is aberrant core fucosylation, which is catalyzed by FUT8 through adding α1,6-fucose to the inner GlcNAc residue of N-glycans [6]. The expression of FUT8 is typically increased in malignant cancers including CRC [7], non-small cell lung cancer [8], triple-negative breast cancer [9], and melanoma [10]. It plays important roles in tumor microenvironment [11] through the regulation of B7-H3 [9], programmed cell death 1 (PDCD1, PD-1) [12], transforming growth factor beta receptor 1 (TGFBR1) [8], epidermal growth factor receptor (EGFR) [13], etc.

B7-H3, a key ICM [14], is frequently upregulated in cancers and involved in promoting migration and invasion, endothelial-to-mesenchymal transition [15], angiogenesis, chemoresistance, and immune evasion, and also affecting tumor cell metabolism [16]. As such, therapeutic strategies targeting B7-H3, such as small-molecule inhibitors, blocking antibodies, bispecific antibodies, chimeric antigen receptor T cells, and combination therapies, have already been demonstrated efficacy in various cancer settings [17]. B7-H3 is highly N-glycosylated in cancers [18], which has been observed to be degraded by FUT8 inhibition in triple-negative breast cancer [9]. However, the mechanisms underlying the degradation of afucosylated B7-H3 are still unclear.

The proteolysis occurs through two main mechanisms, the ubiquitin-proteasomal pathway and the autophagy-lysosomal pathway [19]. Autophagy is a process in which proteins are degraded in lysosomes and is typically divided into three categories including macroautophagy, microautophagy, and CMA. Contrary to macroautophagy and microautophagy, in which the proteins are transfered to the inside vesicles of lysosomes, CMA substrate proteins are recognized by the cytosolic chaperone HSC70 one-by-one, which then takes them to the surface of lysosome together with modulatory co-chaperones [20]. All CMA substrate proteins carry a specific sequence (KFERQ-like motif), which can be recognized by HSC70. Following this targeting phase, the substrate engages with the cytosolic tail of the receptor for the CMA pathway, lysosome-associated membrane protein type 2 A (LAMP2A) [21]. Here, we discovered that B7-H3 was defucosylated by inhibition of FUT8 and consequently was recognized by HSC70, which impelled autophagy-lysosomal proteolysis of B7-H3 through CMA pathway in CRC cells.

Given the critical roles of FUT8 in cancer biology, it is poised to be a druggable target for cancer treatment as summarized in our recent review [22]. A conventional strategy is to develop natural substrate guanosine diphosphate fucose (GDP-Fuc) analogs, such as 2-fluoro-L-fucose (2F-Fuc) and GDP-2F-Fuc, which could competitively inhibit FUT8 activity. While, these analogs are either high polary or low stability, which make their use in vivo limited for poor penetration of the cell membrane. Other fucosylation inhibitors including SGN-2FF, Fucotrim I (PD-Rha6F2-1P) and Fucotrim II (PD-Rha6F3-1P) straightly targeting de novo GDP-fucose biosynthesis via competitive GDP-mannose-4,6-dehydratase inhibition, were discovered recently, while there is no evidence supporting that they could inhibit specific FUTs selectively such as FUT8. Although these intensive efforts have focused on inhibition of FUT8, the studies were hindered by the insufficient information of FUTs structure, particularly the structure of the complex with the substrate of FUTs. Therefore, it is imperative to discover novel FUT8 inhibitors with GDP-Fuc independent structure that disrupt the combination of GDP and FUT8 for CRC treatment [22].

Considering the difficulties in the development of small-molecular FUT8 inhibitors, we first searched the most probable conformation of the binding site to identify the key residues on FUT8 structure by employing molecular dynamics (MD) simulations, and calculations and experiments based on mutated molecular mechanics/generalized Born surface area (MM/GBSA). The results demonstrated that the residues Arg365, Lys369, Tyr250, Ser469, Gln470, Gly221, Tyr220, and Asp453 were the most important binding determinants for the interaction between small-molecule and FUT8 protein. Then we developed a hierarchical strategy that combines virtual screening, bioassays, and chemical optimization to identify FDW028 as a novel potential inhibitor for FUT8. Consequent binding assays and target affinity experiments confirmed that FDW028 perfectly bound to FUT8. As expected, FDW028 showed promising anti-CRC effects both in vitro and in vivo. Moreover, FDW028-mediated defucosylation was also validated to promote the CMA degradation of B7-H3. These findings further revealed the oncogenic roles of FUT8 and provided compelling evidence to support FDW028 as a potent anti-CRC agent by targeting FUT8.

## Materials and methods

### Human cohorts

A total of 192 human CRC tissues were collected from the Second Affiliated Hospital of Soochow University. The tissues were stained with hematoxylin-eosin (HE) and confirmed by pathologists. The tissue-derived patients did not receive chemotherapy or radiotherapy before surgery. All patients signed the informed consent. The procedures performed in the studies were in accordance with the ethical standards of the ethics committee of Soochow University (No. IRB-202008-A260) and with the 1964 Helsinki declaration and its later amendments or comparable ethical standards.

### Immunohistochemistry (IHC)

For IHC, the tissue chips were deparaffinized in xylene, hydrated in ethyl alcohol and washed in tap water. The tissue chips were stained with aspergillus oryzae lectin (AOL), which specifically recognizes core fucose in N-glycans [23], or B7-H3 antibody and diaminobenzidine in an Envision System (Dako, Denmark). Slides were viewed and imaged on a microscope system (Olympus, Japan). Two pathologists performed independent reviews of the IHC results. The stain strength was scored at 0–5, and the stain prevalence was scored at 0 (negative), 1–2 (low) and 3–5 (high).

### Cell lines

The cell lines were all purchased from the American Type Culture Collection. The human CRC cell lines SW480 and HCT-8 cells and murine macrophage cell line MH-S cell were cultured in RPMI 1640 medium (Hyclone) containing 10% fetal bovine serum (FBS, Gibco). The murine CRC cell line Mc38 cells were cultured in DMEM medium (Hyclone) containing 10% FBS. All cells were authenticated by STR profiling and tested for mycoplasma contamination. They were cultured in an incubator (Thermo, USA) with constant temperature of 37 °C and 5% CO_2_.

### Transfection of siRNA and plasmid

SW480 and HCT-8 cells were transfected with siRNA or plasmids using lipofectamine 2000 (Invitrogen, USA) in OptiMEM (Hyclone, USA) according to the manufacturer’s instructions. The siRNAs for FUT8, HSC70, and LAMP2A were purchased from Genepharma (China). The expression plasmids were synthesized by GENEWIZ (China).

### Quantitative PCR

SW480 and HCT-8 cells were transfected with 100 nM siRNAs of FUTs for 48 h. Total RNAs were isolated by using TRIzol (Invitrogen, USA) according to the manufacturer’s instructions and then reversely transcripted into cDNA with random primers (TaKaRa, Japan) and RT-Kit (Thermo, USA). The cDNA was amplified by PCR using primers listed in Table S1.

### Immunoblot and co-immunoprecipitation (co-IP)

SW480 and HCT-8 cells were harvested and lysed in RIPA buffer (Beyotime, China), and protein concentrations were determined by a BCA assay (TaKaRa, Japan). Equal amounts of protein were separated by SDS-PAGE and transferred to polyvinylidene difluoride membranes. After normalization, the blots were probed with appropriate antibodies or Biotin-labeled AOL, then incubated with corresponding HRP-labeled secondary Abs or HRP-conjugated streptavidin. Quantification of blot was performed using ImageJ software. For co-IP, cells were harvested and lysed in lysis buffer. After preclearing Anti-Flag M2 Affinity Gel (Sigma, USA) according to the manufacturer’s instructions, whole-cell lysates were used for co-IP and the mixture was incubated at 4 °C for overnight. Purified B7-H3-Flag protein was eluted under acidic conditions with 0.1 M glycine HCl at pH 2.5. The primary antibodies against B7-H3 (#sc-376769, Santa Cruz Biotech., USA), Lysosomal associated membrane protein 1 (LAMP1) (#sc-20011, Santa Cruz Biotech., USA), β-actin (#AF0003, Beyotime, China), HSC70 (#AF1132, Beyotime, China), mTOR (#AF1648, Beyotime, China), phospho-mTOR (Ser2481; #AF5869, Beyotime, China), Na/K ATPase (#ab76020, Abcam, USA), PD-1 (#367404, BioLegend, USA), PD-L1 (#13684, CST, USA), PD-L2 (#ab200377, Abcam, USA), LAMP2A (#51-2200, Thermo, USA), AKT (#4685, CST, USA), phospho-AKT (Ser473; #4060, CST, USA), and Flag (#M185-3L, MBL Beijing Biotech., China) were employed.

### Cell surface biotinylation

SW480 cells were treated with sulfo-N-hydroxysuccinimide-SS-biotin (1 mg/ml in PBS; Thermo, USA) and lysed with lysis buffer. After centrifugation, the supernatants of lysates were incubated with streptavidin-agarose beads for 1 h at room temperature. After washing, cell surface proteins were recovered from the resin by incubation of the beads with 2 × laemmli buffer containing 100 mM dithiothreitol. Cell membrane proteins were then subjected to SDS-polyacrylamide gel electrophoresis and immunoblot analysis.

### Cell viability assay

SW480 and HCT-8 cells were transfected with 100 nM siRNA for 48 h or not, before the treatment of FDW028 at 0.2, 1.0, 2.0, 5.0, 10, 20, 50, 100 μM for 72 h, respectively.

### Clone formation assay

SW480 and HCT-8 cells were transfected with plasmids or siRNAs for 24 h and then were seeded into six-well plates (300 cells per well) and incubated at 37 °C for 2 weeks. Clones were stained with 0.1% crystal violet.

### Wound-healing assay

SW480 and HCT-8 cells were seeded into six-well plates in confluent monolayers. Then, the cells were transfected with 100 nM siRNA or treated with 50 μM FDW028. Scratch wounds were created using a sterilized tip in 0.5% serum medium. Images were captured at 0, 48 or 72 h after wounding.

### Transwell migration assay

SW480 and HCT-8 cells (1 × 10^5^) were resuspended in 200 µl medium containing 0.1% serum and then added to the upper compartment of migration chambers (Corning, USA). The bottom chamber was filled with 600 µl cell culture medium with 20% FBS as an attractant. After coculture for 72 h, the cells were fixed in 4% paraformaldehyde and stained with 0.1% crystal violet. The stained cells in 8 random fields were counted at × 100 magnification.

### Immunofluorescence

For immunofluorescence, cells were seeded at approximately 50% confluence in 24-well chamber slides (Cellvis, USA) and subsequently were transfected with 100 nM siRNA or treated with 50 μM FDW028. After removal of culture medium, the chamber slides were washed twice with PBS. Then, cells were fixed with 4% formaldehyde for 20 min and washed three times with PBS for 5 min each. Cells were then permeabilized with 0.2% Triton X-100 and blocked in 3% BSA in PBS for 1 h at room temperature. The primary antibodies were diluted in the blocking buffer (1:200) and incubated in chamber slides for 50 min at room temperature. The cells were rinsed by PBS three times for 5 min each. Secondary antibodies were diluted in PBS (1:800) and incubated for 20 min at room temperature, followed by washing three times with PBS for 5 min each. Cells were examined with Zeiss LSM710 confocal microscope (Carl Zeiss, Germany) fitted with a 63 × oil immersion objective or AIR HD25 confocal microscope (Nikon, USA) fitted with a 100 × oil immersion objective. Micrographs were captured by means of confocal software ZEN system (Carl Zeiss) or NIS-Elements Viewer (Nikon). Colocalization analysis was performed using the ImageJ software.

### Animal experiments

Animal protocols were approved by the Institutional Animal Care and Use Committee at Soochow University. All animal experiments were complied with the ARRIVE guidelines and were carried out in accordance with the National Institutes of Health guide for the care and use of Laboratory animals (NIH Publications No. 8023, revised 1978). Athymic male SCID mice (BALB/cNj-Foxn1^nu^/Gpt, Gem Pharmatech, China) at about 4–6 weeks were used to explore the effect of FDW028 on the growth of CRC. Approximately 8 × 10^6^ SW480 cells were collected and mixed with matrigel at a 1:1 ratio by volume and then injected into the lower back region of SCID mice. When tumors were palpable, mice were randomly divided into 5 groups (*n* = 6). One group received vehicle as a control, one group were intravenously administered with 5-Fu at 10 mg/kg, two groups were intravenously administered with FDW028 at 10 or 20 mg/kg, and one group were tumor-aside administered with FDW028 at 10 mg/kg. The tumor sizes and mouse body weights were non-blindly monitored every other day. The tumor size was evaluated according to Eq.: tumor size (mm^3^) = (length × width^2^) × 0.5.

Male mice (C57BL/6 J, Gem Pharmatech, China) at about 4–6 weeks were used to explore the effect of FDW028 on the mCRC. Approximately 1 × 10^6^ Mc38 cells were intravenously engrafted in mice. The mice were randomly divided into 2 groups (*n* = 6). One group received vehicle as a control, and the other group was intravenously administered FDW028 at 20 mg/kg every other day. All mice were non-blindly monitored for survival rates. The lungs of the normal mice and tumor-bearing mice were examined by HE staining.

### MD simulations

The system was neutralized with the counter ions of Na^+^. The whole system was immersed in a rectangular box of TIP3P water molecules, and the water box was extended 12 Å from any solute atom. The particle mesh Ewald (PME) method was employed for the long-range electrostatics [24]. Each system was relaxed by two-stage minimization protocol: first, the protein was fixed, the water molecules and ligand were minimized by 500 cycles of steepest descent and 500 cycles of conjugate gradient minimization; second, the whole system was minimized by 5000 cycles (1000 cycles of steepest descent and 4000 cycles of conjugate gradient minimization). Then, 10 ns MD simulation was performed under a target temperature of 300 K and a target pressure of 1 atm. The SHAKE procedure was employed to constrain all bonds involving hydrogen atoms, and the time step was set to 2 fs. Coordinate trajectories were saved every 10 ps. The MM simulations and MD optimizations were accomplished by using the sander program in AMBER11.

### MM/GBSA binding free energy calculations/decompositions

The binding free energy (ΔG_bind_) of each compound/FUT8 was calculated by the MM/GBSA methodology (see Eq. 1):

$$\begin{array}{*{20}{c}} {{\Delta}G_{{\rm{bind}}}} &= {G_{{\rm{complex}}} - G_{{\rm{protein}}} - G_{{\rm{ligand}}}} \\ {} & { = {\Delta}H + {\Delta}G_{{\rm{solvation}}} - T{\Delta}S} \\ {} & { = {\Delta}E_{\rm{MM}} + {\Delta}G_{\rm{GB}} + {\Delta}G_{\rm{SA}} - T{\Delta}S} \end{array}$$

where ΔE_MM_ represents the gas-phase interaction energy between protein and ligand, which contains the electrostatic (ΔE_ele_) and van der Waals (ΔE_vdw_) terms; ΔG_GB_ and ΔG_SA_ is the polar and non-polar terms of desolvation free energy, respectively [25]. The change of the conformational entropy (-TΔS) was not considered due to high computational cost and low prediction accuracy. The electrostatic solvation energy (ΔG_GB_) was calculated by using the GB model with the parameters developed by Onufriev et al. (igb=2). The exterior dielectric constant was set to 80, and the solute dielectric constant value was set to 1. The non-polar contribution of desolvation (ΔG_SA_) was estimated by solvent accessible surface area using the LCPO method [26]. All energy components were calculated using 100 snapshots extracted from 2.0 to 10 ns. The residue-ligand interaction consists of four parts, van der Waals contribution (ΔG_vdw_), electrostatic contribution (ΔGele), the polar part of desolvation (ΔG_GB_), and the non-polar part of desolvation (ΔG_SA_). The exterior dielectric constant was set to 80, and the solute dielectric constant value was set to 1. The non-polar contribution of desolvation (ΔG_SA_) was calculated by SASA using the ICOSA technique.

### Molecular docking-based virtual screening procedure

The whole protein structure of FUT8 from AlphaFold DB was employed for the simulations [27]. For Glide docking-based virtual screening, the stable and concentrated FUT8 conformation from MD simulation was used as the structural template. First, for FUT8 model, all water molecules were eliminated, the damaged side chains were fixed, and the missing hydrogen atoms were added using the *Protein Preparation Wizard* module of Schrödinger 9.0. The protonation states and partial charges were also then assigned using the *OPLS2005* force field. The binding pocket was produced using the *Receptor Grid Generation* module. The binding pocket was sized at 20 Å × 20 Å × 20 Å and positioned centrally on the centroid of the likely important residues in the FUT8 model active site. Then, TargetMol’s ChemDiv library, which contains more than a million chemicals, was chosen as the screening database. All the small compounds in the ChemDiv database were preprocessed using *LigPrep* mode. The tautomers were generated for each molecule in ChemDiv at pH of 7.0 ± 2.0, and the various combinations of chiralities were produced by setting the maximum number of stereoisomers in *Epik* to 32. Finally, the well-prepared ChemDiv database with more than 2.6 million chemicals was loaded into the Glide docking-based VS process.

### Thermal shift assay

FUT8-Flag protein was firstly purified from SW480 cells by co-IP. The protein solution was diluted with cold PBS and divided into two equal parts, with one part treated with DMSO and the other part treated with FDW028 (200 μM). After incubation at room temperature for 30 min, the two parts were divided into 6 aliquots (50 μl) respectively, then heated at temperatures of 39.0, 42.3, 46.4, 51.9, 56.1 and 59.0 °C for 12 h, respectively. The samples were cooled and kept on ice. Then, each aliquot was analyzed by immunoblot assays.

### Grating-coupled interferometry (GCI)

FUT8 protein was immobilized on a 4PCP-WAVE chip by standard amine coupling chemistry with a flow of 10 μl/min to the desired surface density level. An empty surface, which was activated or deactivated, was used as a reference channel. The running buffer was PBS-P buffer in 3% DMSO (Sigma, USA) at pH 7.5. The kinetic run at 60 µl/min included 20 startup cycles of running buffer injections, followed by a 1:2 dilution series of FDW028 (0.781, 1.526, 3.125, 6.25, 12.5, 25, 50, and 100 μM) with one blank per injection. DMSO calibrations were performed at the end of the series. All the cycles in the kinetic run included baseline for 45 sec, association for 60 sec, and dissociation for 60 sec. Regeneration was not required. Data adjustments including DMSO calibration correction, X-offset correction, and blank subtraction were performed with the Creoptix WAVE control software. Global fitting with bulk correction for both association and dissociation was used to obtain the kinetic rate constants k_a_ and k_d_, as well as the R_max_. Equilibrium analysis included an offset correction.

### Target pulldown

Biotin tags were introduced into FDW028 (FDW028-PEG6 biotin). After SW480 cells transfected with FUT8-Flag plasmids, cells were harvested and lysed. The cell lysates were treated with 0.2 mM FDW028-PEG6-biotin and the mixture was incubated at 4 °C for 1 h. After preclearing streptavidin-coupled Dynabeads (Thermo, USA) according to the manufacturer’s instructions, the mixture mentioned above and Dynabeads was incubated at room temperature for 1 h. After washing, probe-protein complexes were recovered from the resin by incubation of the beads with 2 × laemmli buffer containing 100 mM dithiothreitol. FUT8-Flag in probe-protein complexes was then detected by western blotting and mass spectrometry (MS) determination.

### MS analysis

Probe-protein complexes in target pulldown experiment was freeze-dried, dissolved in Tris and urea buffer, reduced by 20 mM dithiothreitol for 5 min at 100 °C, and then alkylated by 40 mM iodoacetamide for 40 min in the dark at room temperature. Probe-protein complexes was then treated with sequencing grade trypsin (1:25, enzyme-to-substrate) at 37 °C overnight. Digested products were then added with 10% TFA to adjust to acid, desalted with Stag Tip C_18_, dried again and dissolved in 0.1% formic acid.

The desalted samples were re-solubilized using 0.1% formic acid, and appropriate amounts of peptides were taken from each case for chromatographic separation using a nanoliter flow rate Easy-nLC 1200 chromatography system (Thermo Scientific, USA). The sample was separated on a 25 cm in-house packed column (360 μm o.d. × 75 μm I.d.) containing C18 resin (2.2 μm, 100 Å; Michrom Bioresources, USA) at a flow rate of 300 nl/min. Solution A was 0.1% formic acid in water, and solution B was 80% acetonitrile and 0.1% formic acid. The chromatographic column was equilibrated with 100% of liquid A, and 0-1 min is 8–10% solution B, 1–52 min is 10–32% phase B, 52–53 min is 32–95% phase B, and 52–60 min is 95% phase B. The peptides were separated and analyzed by DIA MS using a Q-Exactive HF-X mass spectrometer (Thermo Scientific, USA). For DDA mode, the mass spectrometer selected the “top-20” most abundant precursor ions from a full MS spectrum for subsequent MS/MS fragment analysis. The spray voltage was set to 2.1 kV, the funnel RF level at 40, and the heated capillary at 320 °C. Full MS resolutions were set to 60,000, and the full MS AGC target was 3 × A with a maximum inject time of 30 ms. The mass range was set to 400–1200 m/z. The AGC target value for fragment spectra was set at 1e5, and the resolution threshold was kept at 30,000 with an IT of 50 ms. The isolation width was set at 1.6 m/z. The normalized collision energy was set at 28. Only precursors charged between +2 and +7 that achieved a minimum AGC of 8 × 10^3^ were acquired. Dynamic exclusion was set to 40 s to exclude all isotopes in a cluster.

The MS data were searched using PEAKS online against a human protein database downloaded from the www.uniprot.org. The enzyme was set to trypsin/P with up to 2 missed cleavages. Carbamidomethylation was selected as a fixed modification, while oxidation and acetylation (protein N-term) were selected as variable modifications. A cut-off of 1% FDR was set at peptide spectrum matches, and a cut-off of 1% FDR at the protein level.

### Statistical analysis

All data represent as mean ± SD, unless otherwise stated. Comparisons between two groups were performed using student’s *t*-test. Comparisons among three or more groups were performed using a one-way analysis of variance (ANOVA). The correlation of FUT8 with B7-H3 and clinical parameters was determined using the Pearson’s χ^2^ method; *P* < 0.05 was considered to be statistically significant.

## Results

### FUT8 mediates core fucosylation of B7-H3 in CRC

**Fig. 1:**
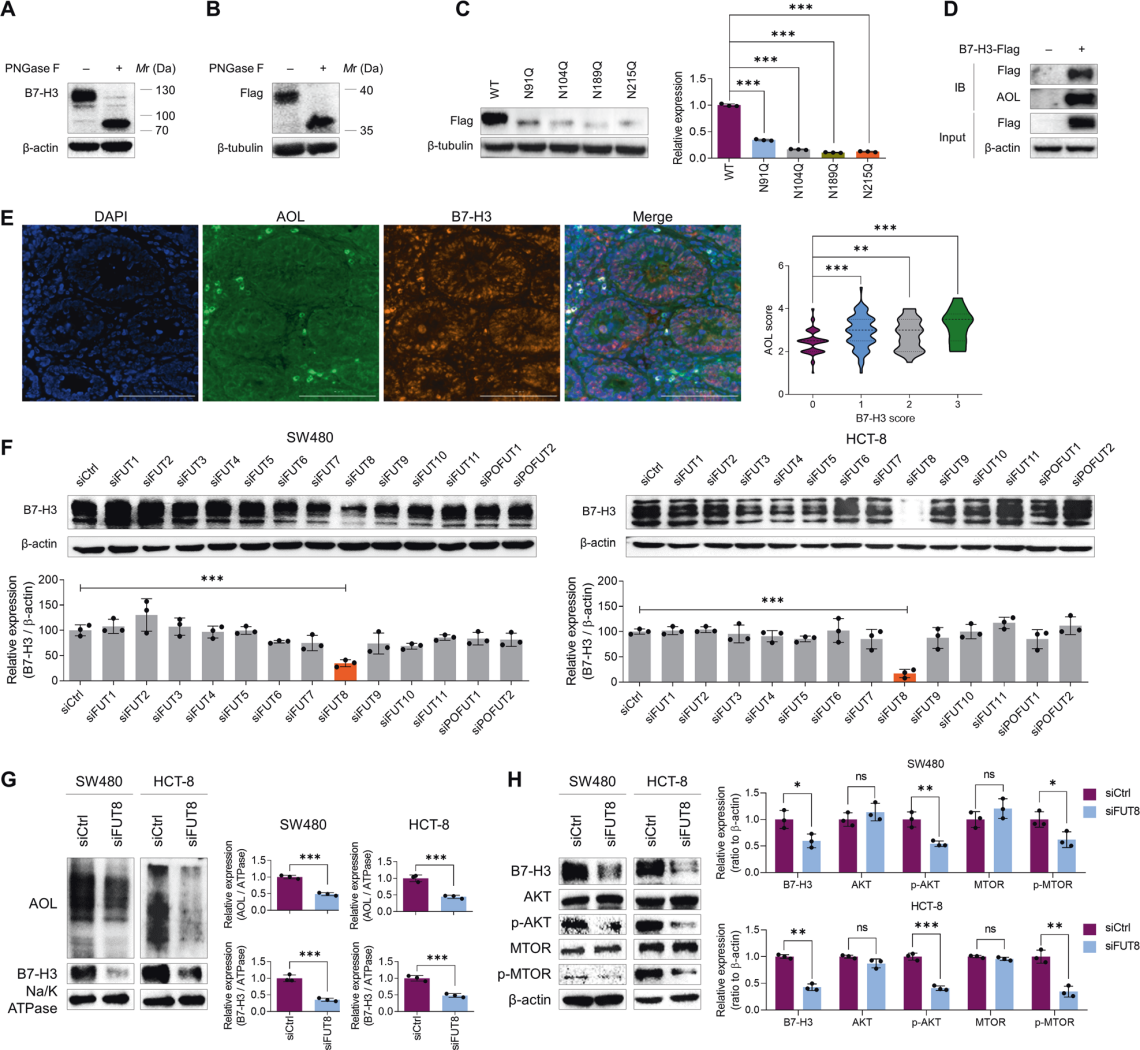
B7-H3 is N-glycosylated and core fucosylated in CRC cells. **A** Immunoblots of B7-H3 treated with or without PNGase F at 37 °C for 3 h. **B** Immunoblots of truncated B7-H3 containing the first IgV-IgC domain (aa1-258) treated with or without PNGase F at 37 °C for 3 h. **C** Immunoblots of truncated B7-H3 containing N91Q, N104Q, N189Q or N215Q mutation. **D** AOL blot of Flag-B7-H3 immunoprecipitated by anti-Flag beads. **E** IHC stains of AOL and B7-H3 in CRC tissues. **F** Immunoblots of B7-H3 in SW480 and HCT-8 cells upon knockdown of each fucosyltransferase. **G** Immunoblots of membranous AOL and B7-H3 in SW480 and HCT-8 cells upon FUT8 knockdown. **H** Immunoblots of B7-H3 and its downstream AKT/MTOR pathway molecules in SW480 and HCT-8 cells upon FUT8 knockdown. All experiments were performed in technical triplicates and are displayed as mean ± s.d.; ns no significance; *P < 0.05, **P < 0.01, ***P < 0.001.

Given the close linkage between FUT8 and B7-H3 [9, 14], which both play significant roles in cancer growth and metastasis, we tend to unravel the roles of FUT8-mediated core fucosylation of B7-H3 in CRC. At first, we confirmed the existence of N-glycosylation and core fucosylation on B7-H3 in CRC. We detected the immunoblots of B7-H3 in CRC cells after the treatment of PNGase F that specially cleaves the whole N-glycans. We found that the immunoblots of B7-H3 was shifted from 100–130 kDa to about 70 kDa (Fig. 1A), suggesting there are extensive N-glycans on B7-H3 in CRC cells. It has been reported that B7-H3 contains a near duplication of IgV-IgC domains (aa 43-235 and aa 246–453) with four N-glycan sites on each domain (N91, N104, N189, and N215 on the first domain; N309, N322, N407, and N433 on the second domain) [18]. We then expressed the first domain of B7-H3 and a gel shift was also observed for the reinforced wildtype B7-H3 after the treatment with PNGase F (Fig. 1B). Moreover, after *N* > Q mutation was generated at each N-glycan site in B7-H3, immunoblots showed gel shift of each mutated fragment, further confirming N-glycans occur at these four sites in SW480 cells (Fig. 1C). We overexpressed B7-H3-Flag in SW480 cells and pulled down it with Flag-labeled beads. The co-IP proteins were detected by immunobloting with AOL. The results demonstrated that core fucosylation existed on the B7-H3 protein (Fig. 1D). Consistently, colocalization of B7-H3 and core fucosylation was detected in the most of CRC tissues by using IHC stains (Fig. 1E). High level of core fucosylation was detected in 140 (72.9%) CRC tissues and was positively correlated with the level of B7-H3 (Fig. 1E). Accordingly, B7-H3 was sharply reduced by the silence of FUT8 instead of the other 12 FUTs in SW480 and HCT-8 cells (Fig. 1F and Fig. S1). Since B7-H3 is a type I transmembrane protein and mainly expressed on the cell membrane, we detected the level of B7-H3 and core fucosylation on the cell membrane upon FUT8 knockdown. We found that both B7-H3 and core fucosylation were evidently reduced by FUT8 knockdown (Fig. 1G). In addition to B7-H3, its downstream AKT serine/threonine kinase (AKT) / mechanistic target of rapamycin kinase (MTOR) signaling pathway was also blocked by the FUT8 knockdown in CRC cells (Fig. 1H).

### Defucosylation induced lysosomal degradation of B7-H3 in CRC

**Fig. 2:**
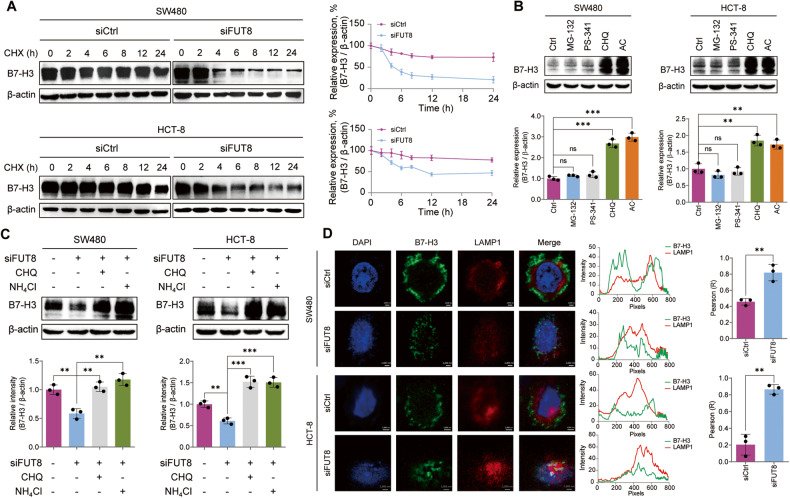
Defucosylation-promoted lysosomal degradation of B7-H3. **A** Immunoblots of B7-H3 protein in SW480 and HCT-8 cells that were treated with 100 μM CHX for the indicated time after FUT8 knockdown or not. **B** Immunoblots of B7-H3 protein in SW480 and HCT-8 cells that were treated with MG-132 (20 μM), PS-341 (50 nM), CHQ (50 μM), and AC (100 μM), respectively. **C** Immunoblots of B7-H3 protein in SW480 and HCT-8 cells that were treated with CHQ (50 μM) or AC (100 μM) after FUT8 knockdown. **D** Immunofluorescence shows the colocalization of B7-H3 and LAMP1 upon FUT8 knockdown. The intensity profiles of B7-H3 and LAMP1 are plotted in the middle panel. The statistical results of colocalization factor (Pearson’s *R* value) are shown on the right panel. All experiments were performed in technical triplicates and are displayed as mean ± s.d.; ns no significance; **P < 0.01, ***P < 0.001.

To explore the roles of core fucosylation in the stability of B7-H3, we treated CRC cells with FUT8 siRNA and followed by cycloheximide (CHX), an inhibitor of protein biosynthesis. We found a faster degradation rate of afucosylated B7-H3 compared with the fucosylated ones (Fig. 2A), suggesting a critical role of core fucosylation in the stability of B7-H3. To find out the mechanism of the defucosylation-mediated degradation of B7-H3, we treated CRC cells with proteasome inhibitors, MG-132 and bortezomib (PS-341), or lysosome inhibitors, chloroquine (CHQ) and ammonium chloride (AC). The immunoblots showed that either CHQ or AC markedly boosted B7-H3, but not MG-132 and PS-341 (Fig. 2B), suggesting that B7-H3 is mainly degraded in lysosome in CRC cells. CHQ or AC also alleviated FUT8 knockdown-mediated inhibition of B7-H3 (Fig. 2C). LAMP1 is distributed among lysosomal organelles and routinely used as a lysosome marker. FUT8 knockdown obviously augmented the colocalization of B7-H3 and LAMP1, demonstrating the locomotion of afucosylated B7-H3 to lysosome (Fig. 2D). These findings consistently demonstrated that FUT8-silence-mediated defucosylation stimulates lysosomal degradation of B7-H3 in CRC cells.

### Defucosylation impelled lysosomal degradation of B7-H3 through CMA pathway

**Fig. 3:**
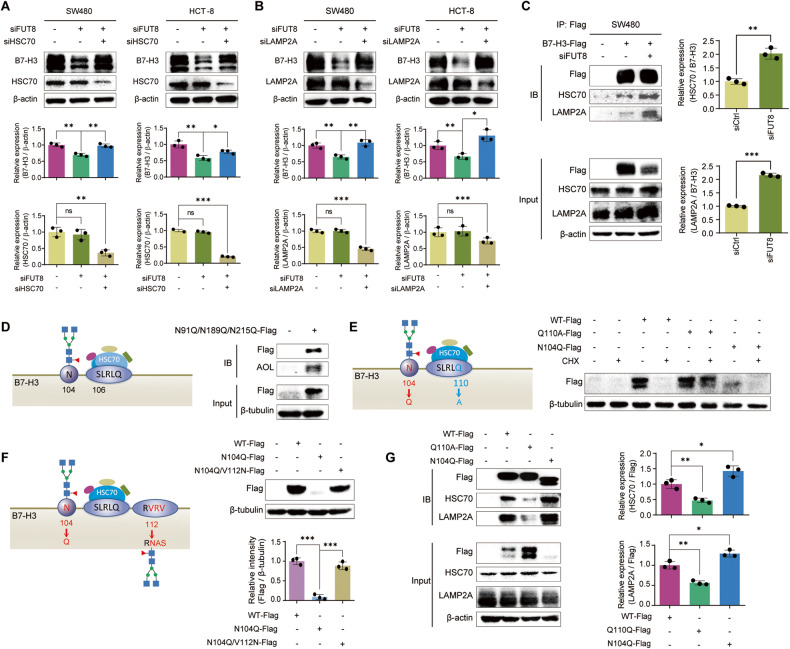
Defucosylation-promoted CMA degradation of B7-H3. **A** Immunoblots of B7-H3 protein in SW480 and HCT-8 cells after knockdown of FUT8 and / or HSC70. **B** Immunoblots of B7-H3 protein in SW480 and HCT-8 cells after knockdown of FUT8 and / or LAMP2A. **C** Co-IP assays showed the effects of FUT8 knockdown on the interaction between B7-H3 and HSC70 or LAMP2A. **D** Schematics of truncated B7-H3 (aa 1-258) with the N104 glycosylation site and 106-110SLRLQ motif for HSC70 binding, and AOL blots of the co-IP B7-H3 protein with wildtype N104 and N91Q/N189Q/N215Q mutations. **E** Schematics of truncated B7-H3 (aa 1-258) with N104Q or Q110A mutation, and immunoblots of truncated B7-H3 in SW480 cells that were treated with 100 μM CHX after transfection of B7-H3 expression plasmids containing N104Q or Q110A mutation. **F** Schematics of truncated B7-H3 (aa 1-258) with N104Q or VRV112-114NAS mutation, and immunoblots of truncated B7-H3 containing N104Q and / or VRV112-114NAS mutation in SW480 cells. **G** Co-IP assays showed the interaction between the truncated B7-H3 containing N104Q or Q110A mutation and HSC70 or LAMP2A. All experiments were performed in technical triplicates and are displayed as mean ± s.d.; ns no significance; *P < 0.05, **P < 0.01, ***P < 0.001.

Since canonical KFERQ-like motifs (SLRLQ at aa 106-110 and aa 324-328) exhibit on B7-H3 [28], we speculated that lysosomal degradation of B7-H3 occurs through CMA pathway. Indeed, the silence of either HSC70 or LAMP2A alleviated the FUT8 knockdown-mediated degradation of B7-H3 in both SW480 and HCT-8 cells (Fig. 3A, B). Co-IP liquid chromatography-tandem mass spectrometry (LC–MS/MS) analysis of the proteins in Fig. 1D led to the identification of HSC70 protein (Table S2), suggesting the intracellular binding of HSC70 with B7-H3. Moreover, co-IP immunoblots showed that much more HSC70 and LAMP2A bound to the afucosylated B7-H3 compared with the fucosylated ones (Fig. 3C). Interestingly, the SLRLQ motifs are close to the N-glycan sites N104 and N322 (Fig. 3D), suggesting that the binding of HSC70 with B7-H3 might be disrupted by the N-glycans. We then constructed a plasmid to express the first IgV-IgC domain with N > Q mutation at N91, N189, and N215 loci. AOL blots showed that core fucosylation existed at N104 locus (Fig. 3E). In case that N104 was replaced by glutamine, a decreased level of aglycosylated fragment with a shorter mobility was detected (Fig. 3E). When the SLRLQ motif was mutated by Q110A, the IgV-IgC fragment became much stable, even after the treatment with CHX (Fig. 3E). To further confirm the impacts of N-glycan on the binding of HSC70, we generated an artificial N-glycan site close to the SLRLQ motif with double mutation of VRV112-114NAS and N104Q. We found that the artificial N-glycan disrupted the binding of HSC70 and resulted in a stable IgV-IgC fragment (Fig. 3F). The co-IP results demonstrated that less HSC70 and LAMP2A bound to the IgV-IgC fragment with Q110A mutation, but much more to the fragment containing N104Q mutation (Fig. 3G). These findings suggest that the lysosomal degradation of B7-H3 occurred through canonical CMA pathway with the binding of HSC70 to 106-110SLRLQ motif, which is hindered by the core fucosylation at N104. Another piece of evidence to support this is that the macroautophagy inhibitor 3-methyladenine (3-MA) shows no effects on the level of B7-H3 in SW480 and HCT-8 cells (Fig. S2).

### Discovery of FUT8 inhibitor FDW028

**Fig. 4:**
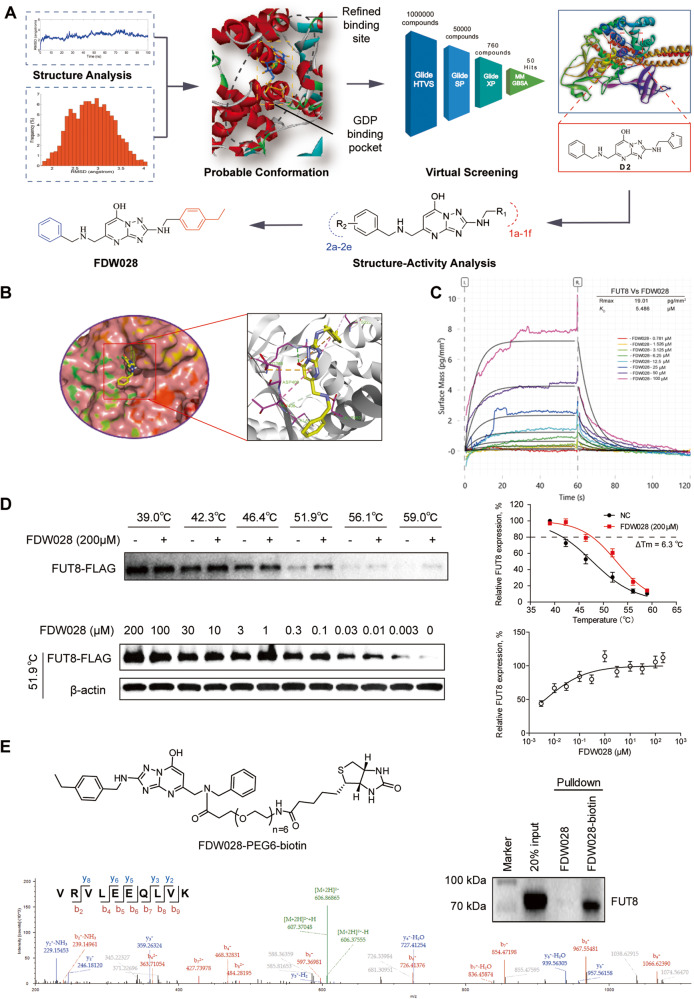
Discovery of FUT8 inhibitor FDW028. **A** Strategies for [1, 2, 4] triazolo [1,5-a] pyrimidin-7-ol skeleton discovery. **B** Docking prediction of FDW028 binding to FUT8. **C** Binding kinetics for FUT8 versus FDW028 obtained from GCI experiments. Sensograms are shown in multiple color with the respective fits in black, and include table summaries of the dissociation constant *K*_D_. **D** Protein thermal shift assay curves for FUT8 with 200 μM FDW028 at gradient temperatures from 39.0 to 59.0 °C (up), or with the indicated concentration of FDW028 at 51.9 °C [45]. **E** The immunoblots (up) and LC–MS/MS spectrum [45] of FUT8 that was pulled down by FDW028-biotin. The structure of FDW028-biotin is also shown.

Considering the limited information of FUT8-acceptor substrate complex structure, it is challenging to develop small-molecule inhibitors targeting FUT8 due to the lack of small-molecule with definite FUT8 binding sites. Despite the fact that few studies have identified potential crucial residues interacting with GDP like Arg365, Lys369, Tyr250, Ser469 and Asp453, a precise small-molecule binding pocket to interdict the combination of GDP and FUT8 remains unknown. Given the difficulties in small-molecule FUT8 inhibitors development, we first searched the most probable conformation of the binding site to find the key residues on FUT8 structure with whole sequence length from AlphaFold protein structure database (AlphaFold DB) by employing MD simulations and MM/GBSA free energy calculations. Then the well-minimized FUT8 structure was determined and the center of mass of the GDP was set as the center of the refined binding site with grid region of 20 Å × 20 Å × 20 Å for molecular docking procedure (Fig. 4A). Based on the refined binding conformation of FUT8 structure, we developed a hierarchical strategy that combines virtual screening, chemical optimization, and bioassays to screen and find novel potential FUT8 inhibitors. Firstly, we carried out an integrated structure-based virtual screening pipeline for obtaining potential FUT8 inhibitors from ChemDiv small compound library (>1 million small compounds) of TargetMol. Then, 1000 diverse top-ranked compounds were reserved by using a sequential Glide docking-based on three scoring functions (HTVS, SP and XP) and MM/GBSA free energy calculations. Finally, applying REOS filtering, drug-likeness properties prediction, core scaffold clustering and 50 potential FUT8 inhibitors were purchased for biological tests. Among them, 10 of the compounds (VS hit rate >20%) displayed dramatically inhibition activity against FUT8, and D2 was identified as the most potent hit and was chosen for further optimization. To obtain more potent inhibitors, D2 was docked to the binding pocket of FUT8 (Fig. S3). The docking indicated that the [1, 2, 4] triazolo[1,5-a]pyrimidin-7-ol skeleton of D2 occupied the binding pocket and formed a hydrogen bond with Gly221 of FUT8. The triazolo ring also underwent amide-Pi stacking with the Val450 and Val471. Unfortunately, no strong hydrogen bonding was found between the rings on either side and the surrounding residues (Fig. S3). Therefore, D2 was optimized by modifying the thiophene ring and benzene ring (Table S3). To reduce the conflict between thiophene ring of D2 and the surrounding negatively charged area, it was replaced with phenyl substituents and benzene could slightly increase the inhibitory activity of the compound (D2-1a, Table S3). Subsequently, to induce hydrogen bond with Asp453, different substituted benzene rings were introduced to obtain FDW028 (D2-1c, Table S3, Fig. S4) as more potent inhibitor for further binding and functional assays.

### FDW028 targets FUT8 with promising binding affinity and selectivity

FDW028 was docked into the FUT8 site, and the triazolopyrimidinol scaffold of FDW028 formed additional hydrogen bonds with Arg365, Tyr250, and Tyr220. The p-ethyl benzene ring also formed Pi-cation/anion interaction with surrounding residues (Glu373 and Lys369; Fig. 4B), which was consistent with our previous MD simulations results. Subsequently, GCI [29] binding assays were performed at a series concentration of FDW028 (0, 0.781, 1.526, 3.125, 6.25, 12.5, 25, 50 and 100 μM, in triplicates injected in reverse orders) to evaluate the ligand-binding affinity of FDW028 for FUT8. The result revealed a dose-dependent interaction between FDW028 and FUT8 with a *K*_D_ value of 5.486 μM (Fig. 4C). To further validate the interaction between FDW028 and FUT8, the protein thermal shift assay was performed. The result showed that FDW028 at 0.2 mM significantly increased the melting temperature (T_m_) of FUT8 by 6.3 °C (Fig. 4D) and stabilized FUT8 in CRC cells in a dose-dependent manner (Fig. 4D). To profile target engagement and selectivity of FDW028, we synthesized a biotin-labeled FDW028 probe (FDW028-PEG6-biotin; Fig. S5) for chemical pulldown experiments [30]. The cell lysates of SW480 were treated with 0.2 mM FDW028-PEG6-biotin and probe-protein complexes were collected with streptavidin-coupled Dynabeads. FUT8-Flag in probe-protein complexes was then detected by western blotting and LC–MS/MS. The results showed that much more FUT8-Flag was pulled down by FDW028-PEG6-biotin than the control (Fig. 4E). Notably, no FUTs other than FUT8 were pulled down by FDW028-PEG6-biotin, indicating a specific binding of FDW0028 to FUT8. Collectively, these results indicate that FDW028 potently and selectively binds to FUT8.

### FDW028 exerted potent anti-tumor activities against CRC

**Fig. 5:**
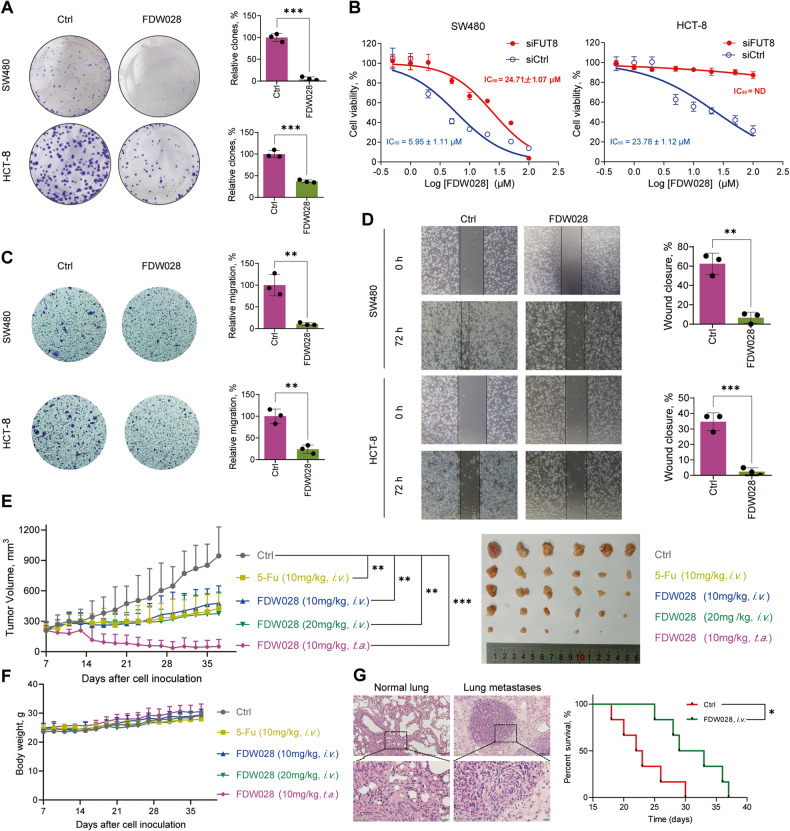
Anti-CRC activities of FDW028. **A** The colony formation abilities of SW480 and HCT-8 cells treated with FDW028 at 50 μM for 14 days. **B** The viabilities of SW480 and HCT-8 cells upon the treatment of FDW028 at the indicated concentrations for 72 h, with or without FUT8 knockdown. Transwell assays (**C**) and wound-healing assays (**D**) for showing the migration abilities of SW480 and HCT-8 cells treated with FDW028 at 50 μM for 72 h. **E** Growth curves and photos of SW480 xenografts treated with FDW028 (10 or 20 mg/kg) or 5-Fu (10 mg/kg). **F** The body weights of tumor-bearing mice with the administration of FDW028 or 5-Fu. **G** The survival of CRPM mice after *i.v.* injection of FDW028 at 20 mg/kg once the other day. HE stains of the lung of normal and tumor-bearing mice are shown. *i.v.* intravenously; *t.a.* tumor-aside; *P < 0.05, **P < 0.01, ***P < 0.001.

To test the anti-tumor activities of FDW028 in CRC, SW480 and HCT-8 cells were treated with FDW028. We found that FDW028 exhibited potent anti-tumor abilities in inhibition of clone formation (Fig. 5A) and cell proliferation with half-maximal inhibitory concentration (IC_50_) values of 5.95 µM and 23.78 µM in SW480 and HCT-8 cells, respectively (Fig. 5B). However, the inhibitory activities of FDW028 were sharply reduced by FUT8 knockdown (Fig. 5B), suggesting that FUT8 is required for FDW028-mediated inhibition of cell proliferation. We then carried transwell migration assays and wound-healing assays to test the effects of FDW028 on the migration capacity of CRC cells. The results showed FDW028 significantly repressed the migration of SW480 and HCT-8 cells (Fig. 5C, D). To confirm the anticancer effectiveness of FDW028 in vivo, the inhibitory roles of FDW028 in SW480 xenografts was tested. The tumor-bearing mice were intravenously administrated with FDW028 every other day. 5-fluorouracil (5-Fu), a clinical first-line remedy for CRC, was utilized as positive control. We found that FDW028 and 5-Fu had a comparable anti-tumor activity in SW480 xenografts (Fig. 5E). Moreover, FDW028 showed an excellent anti-tumor activity with tumor-aside administration (Fig. 5E). Additionally, no significant differences in the body weights of the tumor-bearing mice were observed among the groups (Fig. 5F). Considering that therapeutics for mCRC are urgently required in clinic, we tested the inhibitory roles of FDW028 in Mc38 CRPM mice. We found that intravenous administration of FDW028 apparently prolonged the survival of CRPM mice compared with the control (Fig. 5G). These findings provide plentiful evidence to support FDW028 as a potent anti-mCRC agent.

### FDW028 promoted lysosomal degradation of B7-H3 through CMA pathway

**Fig. 6:**
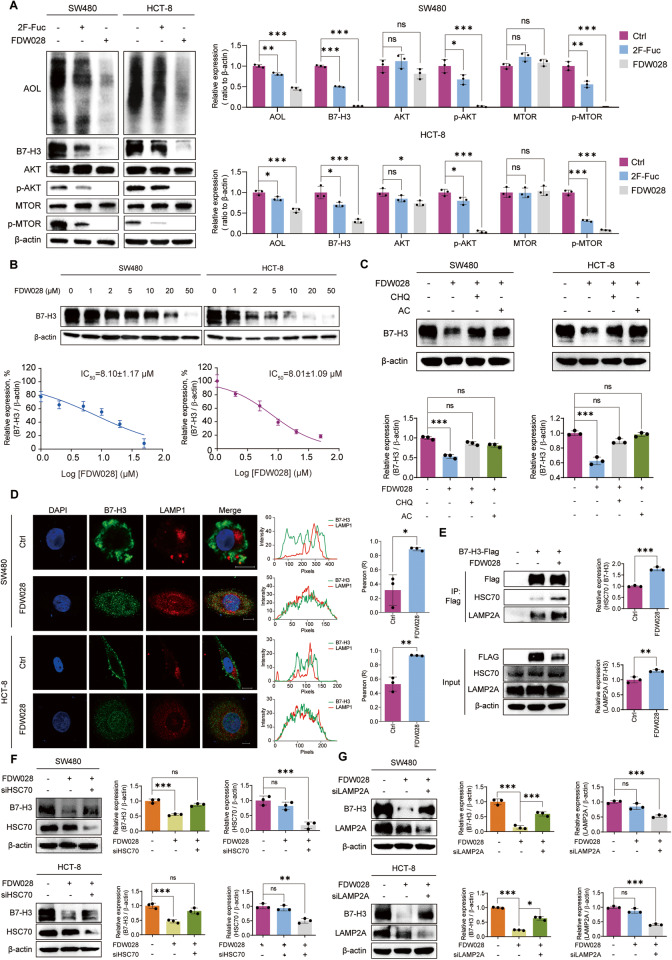
FDW028 promoted defucosylation and CMA degradation of B7-H3. **A** Immunoblots of AOL, B7-H3, and AKT/MTOR pathway molecules in SW480 and HCT-8 cells treated with FDW028 or 2F-Fuc at 50 μM for 72 h. **B** Immunoblots of B7-H3 in SW480 and HCT-8 cells treated with FDW028 at the indicated concentrations for 72 h. **C** Immunoblots of B7-H3 in SW480 and HCT-8 cells after the treatment of FDW028 (50 μM) with or without following CHQ (50 μM) or AC (100 μM). **D** Immunofluorescence shows the colocalization of B7-H3 and LAMP1 upon the treatment of FDW028. The intensity profiles of B7-H3 and LAMP1 are plotted in the middle panel. The statistical results of colocalization factor (Pearson’s R value) are shown on the right panel. **E** Co-IP assays showed the effects of FDW028 on the interaction between B7-H3 and HSC70 or LAMP2A. **F** Immunoblots of B7-H3 in SW480 and HCT-8 cells treated by FDW028 (50 μM) with or without HSC70 siRNA for 72 h. **G** Immunoblots of B7-H3 in SW480 and HCT-8 cells treated by FDW028 (50 μM) with or without LAMP2A siRNA for 72 h. All experiments were performed in technical triplicates and are displayed as mean ± s.d.; ns no significance; *P < 0.05, **P < 0.01, ***P < 0.001.

To confirm the inhibitory roles of FDW028 in the biofunctions of FUT8, we investigated the impacts of FDW028 on core fucosylation and B7-H3 expression in CRC cells. We treated SW480 and HCT-8 cells with FDW028 or 2F-Fuc, a nonselective inhibitor of fucosylation. AOL blots showed that FDW028 markedly attenuated the level of core fucosylation in CRC cells, with a stronger effect than 2F-Fuc (Fig. 6A). Consequently, FDW028 evidently attenuated B7-H3 and blocked its downstream AKT/MTOR signaling pathway (Fig. 6A). Moreover, FDW028 reduced B7-H3 in both SW480 and HCT-8 cells in a concentration-dependent manner (Fig. 6B), which was reversed by CHQ or AC (Fig. 6C), suggesting that FDW028 promoted lysosomal degradation of B7-H3 in CRC cells. We further observed that FDW028 significantly stimulated the lysosomal distribution of B7-H3 (Fig. 6D). The results of co-IP experiments demonstrated that FDW028 apparently boosted the interaction between B7-H3 and HSC70/LAMP2A (Fig. 6E). In addition, FDW028 promoted lysosomal degradation of B7-H3 was interdicted by the silence of either HSC70 (Fig. 6F) or LAMP2A (Fig. 6G). The above findings confirmed that FDW028 attenuated core fucosylation in CRC cells, particularly, promoted the defucosylation and consequent lysosomal degradation of B7-H3, as well as the hindrance of AKT/MTOR pathway.

## Discussion

**Fig. 7:**
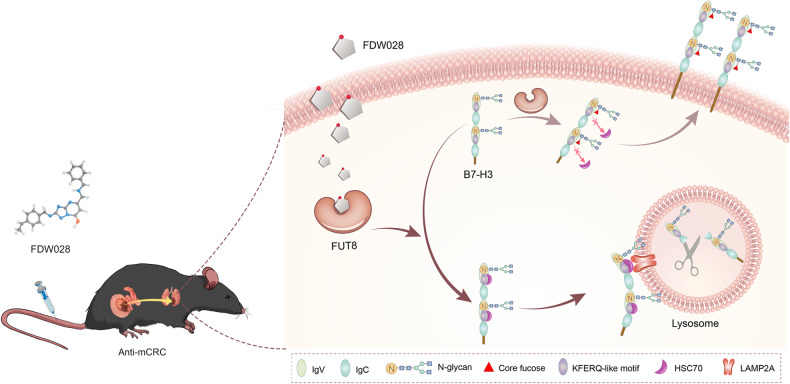
Schematic diagram showing the pharmacological mechanism of FDW028. FDW028, an inhibitor specifically targeted FUT8, promotes defucosylation and consequent HSC70/LAMP2A-mediated lysosomal degradation of B7-H3, and exhibits potent anti-mCRC activities.

FUT8 is the sole FUT catalyzing core fucosylation, an essential N-glycan modification [31]. For this reason, the physiological function of FUT8 is essential, as shown by the fact that Fut8-null mice suffer from death and significant growth retardation during postnatal development [32]. This makes it differ from the other FUTs, functions of which do not correlate with lethality [33]. Even more, FUT8 protein possesses an SH3 domain, an crucial residue for FUT8 activity, which is unique to FUT8 compared with the other glycosyltransferases [34]. Due to the close linkage of FUT8 with cancer [7–13], strenuous efforts have been made to discover FUT8 inhibitors for cancer therapeutics [22]. For instance, SGN-2FF was tested in patients with advanced solid tumors in a phase 1 multicenter clinical trial (NCT# 02952989). However, the study was terminated due to overall benefit and risk profile. One important reason for this may be that SGN-2FF is a nonselective inhibitor of FUT8. In the current study, we discovered an inhibitor, FDW028, potently and selectively targeting FUT8. The high specificity and selectivity of FDW028 for FUT8 was vindicated. FDW028 promoted defucosylation and consequent CMA lysosomal degradation of B7-H3 (Fig. 7). As expected, FDW028 exhibited potent anti-CRC effects both in vitro and in vivo, especially, prolonged the survival of CRPM mice. Furthermore, no significant loss in body weight was observed in FDW028 treatment groups. The efficacy and specificity of FDW028 makes it an exciting new candidate for CRC therapy. Using these data, we can proceed to fully grasp the potential of FUT8 as a critical target in cancer treatment in the future.

Studies have indicated that core fucosylation affects molecules on cell membrane and tumor microenvironment including the ICMs, extracellular matrix and growth factors, and then promotes the progression of cancers [8, 11, 35–37]. For instance, pharmacological inhibition or genetic ablation of FUT8 reduces the expression of ICMs including PD-1, PD-L2, and B7-H3 on cell membrane, thus augmenting T cell activation and resulting in more effective tumor eradication. Consistently, we found that FDW028 apparently attenuated the expression of both PD-L1 and PD-L2 in CRC cells (Fig. S6A), as well as PD-1 in murine macrophages (Fig. S6B). Core fucosylation is critical to the binding of TGFBR1 toward transforming growth factor beta (TGF-β) and thus EMT induced by TGF-β [8]. The phosphorylation of epidermal growth factor receptor (EGFR) induced by EGF is blocked in the embryonic fibroblast cells derived from Fut8-null mice; identical to this, EGF-mediated cellular growth is reduced upon depletion of FUT8 in cancer cells [35]. In Fut8 deficient cells, integrin-mediated cell signaling and migration are inhibited but are partially rescued by the reinforced FUT8, suggesting that FUT8 is vital for the functions of α3β1 integrin [36]. E-cadherin is another substrate of FUT8. Geng et al. found that E-cadherin is decorated with core fucose in highly metastatic lung cancer cells while core fucosylation is absent in lowly metastatic lung cancer cells, showing that cancer metastasis is positively correlated with core fucosylated E-cadherin [37]. However, the regulatory mechanisms of core fucosylation in the expression of glycoproteins are still unclear. Here, we provide plentiful evidence to support that N104 core fucosylation on B7-H3 sterically hinders the binding of HSC70 at 106-110SLRLQ motif, thus preventing CMA lysosomal degradation of B7-H3 and stabilizing membrane B7-H3. Genetic ablation or FDW028-mediated inhibition of FUT8 attenuates membrane B7-H3 through driving the binding of HSC70 to B7-H3 and consequent autophagolysosomal degradation of B7-H3 (Fig. 7). Recently, the posttranslational modifications inclusive of O-GlcNAcylation [38], phosphorylation [39], and acetylation [40] have been revealed to regulate the CMA degradation of substrates. We believe that this is the first work to unveil the core fucosylation mediated regulation of CMA lysosomal proteolysis.

Due to FUT8 being a promising therapeutic target for treating malignant cancer [22], identification of effective and selective FUT8 inhibitors would be a viable strategy for cancer therapy [22, 41]. Up to now, most researches have been focused on the analogs of GDP-Fucose as FUT8 inhibitors [42–44]. However, these analogs of acceptor substrates showed poor specificity toward FUT8 and would inhibit all the fucosylation, while not all the 13 FUTs was involved in tumor progression [10]. Herein, we retrieved the intact FUT8 structure with the whole sequence from AlphaFold DB, through MD simulations and MM/GBSA free energy calculations. We focused on residues and grid region to identify small-molecule binding site of FUT8 for the first time. Thus, we discovered GDP-Fuc independent FUT8 inhibitor FDW028 by a hierarchical strategy composed of docking, virtual screening and bioassays. As far as we know, FDW028 was the first targeted inhibitor of FUT8 ever reported. FDW028 was confirmed to bind to FUT8 as shown in protein thermal shift assays, GCI binding assays, and target affinity experiments, in which no other FUTs presented in the pulldown proteins. FDW028 might bind to Arg365, Tyr250 and Tyr220 of FUT8, where Arg365 is the key active site of FUT8 and contributes to binding and catalysis [34, 41]. Probably due to these reasons, FDW028 showed better properties in affinity, selectivity, and anti-tumor activity compared with 2F-Fuc.

In summary, we discovered a novel FUT8 inhibitor, FDW028, with potent anti-CRC activities. FDW028-mediated defucosylation was validated for the first time to promote the CMA degradation of B7-H3. Therefore, our study further demonstrated the oncogenic roles of FUT8 and provided solid evidence to support FDW028 as an anti-CRC agent by targeting FUT8. In addition to CRC, FUT8 is highly expressed and plays critical roles in the malignant cancers [8–10], suggesting that the anti-tumor activities of FDW028 in these cancers required further investigations. Additionally, the [1, 2, 4] triazolo [1,5-a] pyrimidin-7-ol skeleton in FDW028 provided further discussion on structural optimization and biological evaluation of FUT8 inhibitors. Whatsoever, the discoveries outlined here have made considerable advancements in the development of therapeutics for advanced CRC.

## Supplementary information

aj-checklist

Supplementary Materials

## Data Availability

All data needed to evaluate the conclusions in the paper are present in the paper and/or the Supplementary Materials. Additional data related to this paper may be requested from the authors.
