# Supplementary material for: FDW028, a novel FUT8 inhibitor, impels lysosomal proteolysis of B7-H3 via chaperone-mediated autophagy pathway and exhibits potent efficacy against metastatic colorectal cancer
Source: Cell Death Dis. 2023 Aug 3;14(8):495. doi: 10.1038/s41419-023-06027-0 (PMC10400579; doi:10.1038/s41419-023-06027-0)
Supplement: Supplementary file 2 — Supplementary Materials [file 41419_2023_6027_MOESM2_ESM.pdf]

Supplementary materials

Methods

Synthesis of series of the derivatives. All reagents in this paper were purchased from commercial sources and used as received. Bruker AV-400 instrument and Nuclear Magnetic Resonance Spectrometer DD2-600M were utilized to measure all ^{1}H NMR and ^{13}C NMR spectra. Deuterated solvents were used to dissolve all compounds with tetramethylsilane (TMS) as internal standard. High resolution electrospray ionization (ESI) mass analysis was recorded by Waters Xevo G2-XS Tof high resolution mass spectrometer. LC-ESI-MS analysis was recorded by Agilent 1260-6120 Liquid chromatograph mass spectrometer. The compounds were separated using 200-300 mesh column chromatography silica gel, which was produced by Qingdao Ocean Chemical Works. The amount of silica gel was 50-100 times of the amount of the separated samples. The whole elution process was tracked by thin layer chromatography (TLC). The silica gel 60 GF254 produced by Qingdao Ocean Chemical Works was used for TLC. The silica gel 60 GF254 was detected by ultraviolet radiation at 254 nm wavelength. The integrated synthetic scheme was shown in scheme 1-4.

1. Synthesis of compound D2-1a-f.

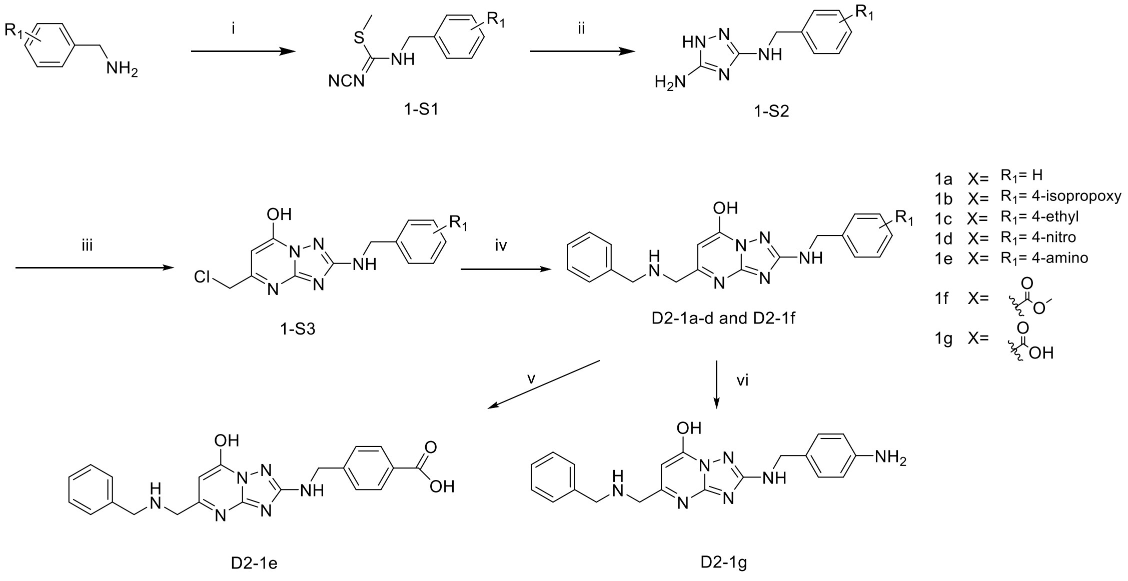

Scheme 1. General Synthesis Methods of compound D2-1a-f.

(i) dimethyl cyanocarbonimidodithioate, Isopropyl alcohol, r.t., 1h; (ii) Hydrazinium hydroxide,

EtOH, 80 °C, 4 h; (iii) Ethyl-4-chloroacetoacet-ate, AcOH, 90 °C, 10h; (iv) Phenylmethanamine, 90 °C, 0.5h; (v) potassium hydroxide, water and tetrahydrofuran, r.t., 2h; (vi) Zn powder and NH_4Cl, MeOH and DMF, 80 °C, 6h.

2-(benzylamino)-5-((benzylamino)methyl)-[1,2,4]triazolo[1,5-a]pyrimidin-7-ol (D2-1a).

Step 1: To a stirred solution of dimethyl cyanocarbonimidodithioate (0.292 g, 2.00 mmol) in IPA (15 mL) was added phenylmethanamine (0.214 g, 2.00 mmol). After stirring for 1 h at room temperature, a precipitate formed. Then the precipitate was collected by filtration and washed with cold ethyl acetate, then pentane and was dried under reduced pressure to afford pure **1a-S1** (0.350 g, 85%) as white solid. ^{1}H NMR (400 MHz, DMSO) δ 8.89 (s, 1H), 7.35 (dd, *J* = 9.8, 4.9 Hz, 2H), 7.29 (d, *J* = 7.2 Hz, 3H), 4.49 (d, *J* = 4.5 Hz, 2H), 2.62 (s, 3H).

Step 2: To a solution of **1a-S1** (0.350 g, 1.71 mmol) in EtOH (50 mL) was added Hydrazinium hydroxide (0.259 g, 5.13 mmol). After stirring for 4 h at 80 °C. The mixture was diluted with H2O (15mL) and extracted with EA (3 × 8 mL), and the combined organic phases were washed with brine, dried over Na2SO4, filtered, concentrated *in vacuo* and crystallized by ethanol to give **1a-S2** (0.284 g, 88%) as white solid. ^{1}H NMR (400 MHz, DMSO-d_6) δ 7.37-7.24 (m, 4H), 7.22-7.16 (m, 1H), 6.34-5.96 (m, 1H), 4.22 (d, *J* = 6.2 Hz, 2H), 1.24 (d, *J* = 3.1 Hz, 1H).

Step 3: To a solution of **1a-S2** (0.284 g, 1.5 mmol) in AcOH (30 mL) was added Ethyl-4-chloroacetoacet-ate (0.295 g, 1.8 mmol). After stirring for 10 h at 90 °C, the reaction mixture was cooled to room temperature and cautiously poured over ice water, obtaining a precipitate that was filtered. The precipitate was crystallized by AcOH to give **1a-S3** (0.195 g, 68%) as white solid. ^{1}H NMR (400 MHz, DMSO-d_6) δ 7.36-7.32 (m, 3H), 7.30 (d, *J* = 7.8 Hz, 1H), 7.26-7.19 (m, 1H), 6.01 (d, *J* = 1.5 Hz, 1H), 4.59 (s, 2H), 4.41 (d, *J* = 5.1 Hz, 2H).

Step 4: A 10 mL round bottom flask was charged with **1a-S3** (0.100 g, 0.34 mmol) and phenylmethanamine (1.5 mL). After stirring for 0.5h at 90 °C, the reaction mixture was cooled to room temperature and cautiously poured over ethyl acetate. Petroleum ether was added to the reaction mixture until a precipitate is formed. The precipitate was

crystallized by EtOH to afford **D2-1a** (0.072 g, 58%) as white solid. ^{1}H NMR (600 MHz, DMSO-d_6) δ 7.57 (d, J = 6.9 Hz, 2H), 7.41 (d, J = 7.0 Hz, 3H), 7.35 (d, J = 7.7 Hz, 2H), 7.31 (t, J = 7.5 Hz, 2H), 7.23 (d, J = 7.3 Hz, 1H), 6.11 (s, 1H), 4.42 (d, J = 6.1 Hz, 2H), 4.21 (s, 2H), 4.11 (s, 2H).

5-((benzylamino)methyl)-2-((4-isopropoxybenzyl)amino)-[1,2,4]triazolo[1,5-a]pyrimidin-7-ol (D2-1b). The title compound was synthesized using the same procedure as that used for **D2-1a** except that (4-isopropoxyphenyl)methanamine was used instead of phenylmethanamine. White solid; yield 35%.^{1}H NMR (400 MHz, DMSO-d_6) δ 7.52-7.45 (m, 2H), 7.34 (dd, J = 2.3, 5.0 Hz, 3H), 7.20-7.13 (m, 2H), 6.81-6.74 (m, 2H), 6.03 (s, 1H), 4.48 (p, J = 6.0 Hz, 1H), 4.25 (d, J = 4.3 Hz, 2H), 4.13 (s, 2H), 4.02 (s, 2H), 1.15 (d, J = 6.0 Hz, 6H).

5-((benzylamino)methyl)-2-((4-ethylbenzyl)amino)-[1,2,4]triazolo[1,5-a]pyrimidin-7-ol (D2-1c, FDW028). The title compound was synthesized using the same procedure as that used for **D2-1a** except that (4-ethylphenyl)methanamine was used instead of phenylmethanamine. White solid; yield 37%.^{1}H NMR (600 MHz, DMSO-d_6) δ 7.55 (d, J = 7.0 Hz, 2H), 7.41 (d, J = 7.3 Hz, 2H), 7.31 (d, J = 6.5 Hz, 1H), 7.25 (d, J = 7.7 Hz, 2H), 7.14 (d, J = 7.7 Hz, 2H), 6.07 (s, 1H), 4.37 (d, J = 6.1 Hz, 2H), 4.19 (s, 2H), 4.07 (s, 2H), 2.56 (q, J = 7.6 Hz, 2H), 1.14 (t, J = 7.6 Hz, 3H). ^{13}C NMR (151 MHz, DMSO-d_6) δ 155.32, 150.65, 142.60, 137.50, 132.47, 130.50, 129.31, 129.01, 128.00, 127.55, 101.58, 50.39, 47.50, 45.72, 28.27, 16.19. HRMS (ESI) m/z: calculated for $C_{22}H_{24}N_6O$ [M + H]$^+$ 389.2084 found 389.2089.

5-((benzylamino)methyl)-2-((4-nitrobenzyl)amino)-[1,2,4]triazolo[1,5-a]pyrimidin-7-ol (D2-1d). The title compound was synthesized using the same procedure as that used for **D2-1a** except that (4-nitrophenyl)methanamine was used instead of phenylmethanamine. White solid; yield 32%.^{1}H NMR (400 MHz, DMSO-d_6) δ 8.24-8.17 (m, 2H), 7.62-7.59 (m, 2H), 7.58-7.54 (m, 2H), 7.43 (dd, J = 2.1, 5.1 Hz, 3H), 6.11 (s, 1H), 4.55 (d, J = 5.7 Hz, 2H), 4.22 (s, 2H), 4.12 (s, 2H).

2-((4-aminobenzyl)amino)-5-((benzylamino)methyl)-[1,2,4]triazolo[1,5-a]pyrimidin-7-ol (D2-1e). Add Zn powder (0.110 g, 1.7 mmol) and NH_4Cl (0.107 g, 2 mmol) to a suspension of **D2-1d** (0.100 g, 0.24 mmol) in MeOH (7 mL) and DMF (1 mL). Sonicate the

reaction for 5 minutes. Stir the reaction mixture at 80 °C for 6 hours. Remove the ZnO by filtration. Wash the ZnO extensively with MeOH. Concentrate the filtrate. Suspend the filtrate in DCM (5 mL) and stir until a precipitate formed. Collect the precipitate. Dry the precipitate in vacuo. White solid; yield 55%. ^{1}H NMR (400 MHz, DMSO-d_6) δ 7.44-7.21 (m, 5H), 6.98 (d, *J* = 8.1 Hz, 2H), 6.49 (d, *J* = 7.9 Hz, 2H), 5.44 (s, 1H), 4.90 (s, 2H), 4.20 (d, *J* = 5.3 Hz, 2H), 3.38 (s, 2H).

Methyl 4-(((5-((benzylamino) methyl)-7-hydroxy-[1,2,4]triazolo[1,5-a]pyrimidin-2-yl) amino)methyl)benzoate (D2-1f). The title compound was synthesized using the same procedure as that used for **D2-1a** except that methyl 4-(aminomethyl)benzoate was used instead of phenylmethanamine. White solid; yield 29%.^{1}H NMR (400 MHz, DMSO-d_6) δ 7.94-7.89 (m, 2H), 7.54 (dd, *J* = 2.3, 7.3 Hz, 2H), 7.49 (d, *J* = 1.8 Hz, 1H), 7.47 (s, 1H), 7.43 (dq, *J* = 2.4, 2.9, 4.7 Hz, 3H), 6.09 (s, 1H), 4.50 (d, *J* = 6.0 Hz, 2H), 4.22 (s, 2H), 4.10 (s, 2H), 3.83 (s, 3H).

4-(((5-((benzylamino)methyl)-7-hydroxy-[1,2,4]triazolo[1,5-a]pyrimidin-2-yl)amino)methyl)benzoic acid (D2-1g). Add **D2-1f** (0.100 g, 0.24 mmol) to a mixture of potassium hydroxide (0.336 g, 6 mmol) in 6 mL of water and 2 mL of tetrahydrofuran. Stir the resulting suspension at room temperature for 2 h. Concentrate the solution by rotary evaporation to remove tetrahydrofuran and dilute to 20 mL volume with water. Take the mixture to pH 2 with concentrated hydrochloric acid. Dry the solid obtained in vacuo. White solid; yield 80%. ^{1}H NMR (400 MHz, DMSO-d_6) δ 7.89 (d, *J* = 8.2 Hz, 2H), 7.53 (dd, *J* = 2.2, 7.4 Hz, 2H), 7.47-7.41 (m, 5H), 6.07 (s, 1H), 4.49 (d, *J* = 6.1 Hz, 2H), 4.21 (s, 2H), 4.08 (s, 2H).

2. Synthesis of compound D2-2a-e.

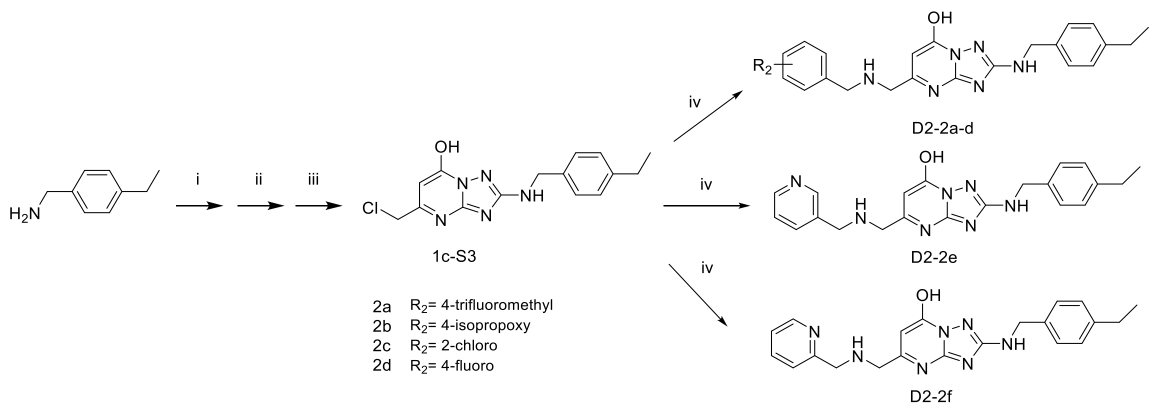

Scheme 2. General Synthesis Methods of compound D2-2a-e. (i) dimethyl cyanocarbonimidodithioate, Isopropyl alcohol, r.t., 1h; (ii) Hydrazinium hydroxide, EtOH, 80 °C, 4 h; (iii) Ethyl-4-chloroacetoacet-ate, AcOH, 90 °C, 10h; (iv) Phenylmethanamine, 90 °C, 0.5h

2-((4-ethylbenzyl)amino)-5-(((4-(trifluoromethyl)benzyl)amino)methyl)-[1,2,4]triazolo[1,5-a]pyrimidin-7-ol(D2-2a).

Step1: 1c-S3 was synthesized using similar procedure as that used for **1a-S3**. **1c-S3** was obtained as white solid (188 mg, 66%). ^{1}H NMR (400 MHz, DMSO-d_6) δ 7.24 (d, *J* = 8.0 Hz, 2H), 7.17-7.11 (m, 2H), 6.01 (s, 1H), 4.58 (s, 2H), 4.35 (d, *J* = 6.3 Hz, 2H), 2.56 (q, *J* = 7.6 Hz, 2H), 1.15 (t, *J* = 7.6 Hz, 3H).

Step2: a 10 mL round bottom flask was charged with **1c-S3** (0.100 g, 1.89 mmol) and (4-(trifluoromethyl) phenyl)methanamine (1.5 mL). After stirring for 0.5h at 90 °C, the reaction mixture was cooled to room temperature and cautiously poured over ethyl acetate. Petroleum ether was added to the reaction mixture until a precipitate is formed. The precipitate was crystallized by EtOH to afford **D2-2a** (0.065 g, 48%) as grey solid. ^{1}H NMR (400 MHz, DMSO-d_6) δ 7.81 (s, 4H), 7.26 (d, *J* = 8.0 Hz, 2H), 7.15 (d, *J* = 8.0 Hz, 2H), 6.12 (s, 1H), 4.38 (d, *J* = 5.8 Hz, 2H), 4.31 (s, 2H), 4.11 (s, 2H), 2.56 (q, *J* = 7.6 Hz, 2H), 1.15 (t, *J* = 7.6 Hz, 3H).

4-((((2-((4-ethylbenzyl)amino)-7-hydroxy-[1,2,4]triazolo[1,5-a]pyrimidin-5-yl)methyl)amino)methyl)benzonitrile (D2-2b). The title compound was synthesized using the same procedure as that used for **D2-2a** except that methyl 4-(aminomethyl)benzoate was used instead of (4-(trifluoromethyl)phenyl)methanamine. White solid; yield 33%.^{1}H NMR (400 MHz, DMSO-d_6) δ 9.99 (s, 1H), 7.98-7.88 (m, 2H), 7.80-7.72 (m, 2H), 7.26 (d, *J* = 8.0 Hz, 2H), 7.15 (d, *J* = 7.9 Hz, 2H), 6.10 (s, 1H), 4.38 (d, *J* = 4.2 Hz, 2H), 4.31 (s, 2H), 4.10 (s, 2H), 2.57 (q, *J* = 7.6 Hz, 2H), 1.15 (t, *J* = 7.6 Hz, 3H).

5-(((2-chlorobenzyl)amino)methyl)-2-((4-ethylbenzyl)amino)-[1,2,4]triazolo[1,5-a]pyrimidin-7-ol (D2-2c). The title compound was synthesized using the same procedure as that used for **D2-2a** except that methyl (2-chlorophenyl)methanamine was used instead of (4-(trifluoromethyl)phenyl)methanamine. White solid; yield 34%.^{1}H NMR (400 MHz, DMSO-d_6) δ 7.56 (dd, *J* = 1.8, 7.5 Hz, 1H), 7.42 (dd, *J* = 1.6, 7.7 Hz, 1H), 7.36 – 7.27 (m,

2H), 7.24 (d, *J* = 8.0 Hz, 2H), 7.16 – 7.11 (m, 2H), 7.00 (t, *J* = 6.4 Hz, 1H), 5.81 (s, 1H), 4.34 (d, *J* = 6.3 Hz, 2H), 3.81 (s, 2H), 3.66 (s, 2H), 2.56 (q, *J* = 7.6 Hz, 2H), 1.15 (t, *J* = 7.6 Hz, 3H).

2-((4-ethylbenzyl)amino)-5-(((4-fluorobenzyl)amino)methyl)-[1,2,4]triazolo[1,5-a]pyrimidin-7-ol (D2-2d). The title compound was synthesized using the same procedure as that used for **D2-2a** except that pyridin-3-ylmethanamine was used instead of (4-fluorophenyl)methanamine. White solid; yield 33%.^{1}H NMR (400 MHz, DMSO-d_6) δ 7.49 – 7.41 (m, 2H), 7.24 (d, *J* = 8.0 Hz, 2H), 7.21 – 7.14 (m, 2H), 7.12 (d, *J* = 7.9 Hz, 2H), 6.81 (t, *J* = 6.4 Hz, 1H), 5.67 (s, 1H), 4.34 (d, *J* = 6.3 Hz, 2H), 3.84 (s, 2H), 3.68 (s, 2H), 2.55 (q, *J* = 7.6 Hz, 2H), 1.15 (t, *J* = 7.6 Hz, 3H).

2-((4-ethylbenzyl)amino)-5-(((pyridin-3-ylmethyl)amino)methyl)-[1,2,4]triazolo[1,5-a] pyrimidin-7-ol (D2-2e). The title compound was synthesized using the same procedure as that used for **D2-2a** except that pyridin-3-ylmethanamine was used instead of (4-(trifluoromethyl)phenyl)methanamine. White solid; yield 36%.^{1}H NMR (400 MHz, DMSO-d_6) δ 8.56 (d, *J* = 2.2 Hz, 1H), 8.47 (dd, *J* = 1.7, 4.8 Hz, 1H), 7.79 (dt, *J* = 2.0, 7.9 Hz, 1H), 7.36 (dd, *J* = 4.8, 7.8 Hz, 1H), 7.24 (d, *J* = 7.9 Hz, 2H), 7.13 (d, *J* = 8.0 Hz, 2H), 6.89 (s, 1H), 5.73 (s, 1H), 4.34 (d, *J* = 6.3 Hz, 2H), 3.81 (s, 2H), 3.65 (s, 2H), 2.56 (q, *J* = 7.6 Hz, 2H), 1.15 (t, *J* = 7.6 Hz, 3H).

2-((4-ethylbenzyl)amino)-5-(((pyridin-2-ylmethyl)amino)methyl)-[1,2,4]triazolo[1,5-a] pyrimidin-7-ol (D2-2f). The title compound was synthesized using the same procedure as that used for **D2-2a** except that pyridin-2-ylmethanamine was used instead of (4-(trifluoromethyl) phenyl)methanamine. White solid; yield 38%^{1}H NMR (400 MHz, DMSO-d_6) δ 8.57 – 8.50 (m, 1H), 7.78 (td, *J* = 1.8, 7.7 Hz, 1H), 7.46 (d, *J* = 7.9 Hz, 1H), 7.29 (dd, *J* = 5.0, 7.5 Hz, 1H), 7.25 (d, *J* = 7.8 Hz, 2H), 7.13 (d, *J* = 7.8 Hz, 2H), 6.80 (t, *J* = 6.4 Hz, 1H), 5.69 (s, 1H), 4.36 (d, *J* = 6.3 Hz, 2H), 3.97 (s, 2H), 3.74 (s, 2H), 2.55 (q, *J* = 7.6 Hz, 2H), 1.15 (t, *J* = 7.6 Hz, 3H).

3. Synthesis of compound D2-3a-b and D2-4a.

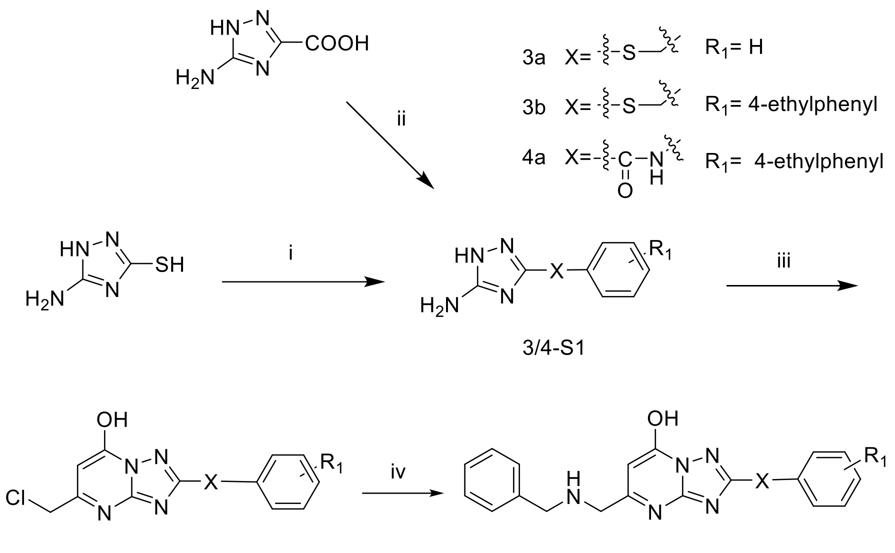

Scheme 3. General Synthesis Methods of compound D2-2a-e. (i) (chloromethyl)benzene, K_2CO_3 and KI, acetone, r.t., 4h; (ii) EDCI, HOBt, DIPEA, DMF, r.t., overnight; (iii) Ethyl-4-chloroacetoacet-ate, AcOH, 90 °C, 10h; (iv) Phenylmethanamine, 90 °C, 0.5h

5-((benzylamino)methyl)-2-(benzylthio)-[1,2,4]triazolo[1,5-a]pyrimidin-7-ol (D2-3a).

Step 1: A solution of 5-amino-1H-1,2,4-triazole-3-thiol (0.393 g, 3.00 mmol), chloromethylbenzene (0.38 ml, 3.30 mmol), Na_2CO_3 (0.474 g, 4.50 mmol) and KI (0.048 g, 0.30 mmol) in acetone (12 mL) was stirred at room temperature for 4 h. The mixture was diluted with H_2O (20 mL) and extracted with EA (3 × 10 mL), and the combined organic phases were washed with brine, dried over Na_2SO_4, filtered, and concentrated. Moreover, purifying with flash chromatography (20% MeOH in DCM) gives **3a-S1** as a white solid (0.390 g, 63%).^{1}H NMR (400 MHz, DMSO-d_6) δ 11.93 (s, 1H), 7.40-7.33 (m, 2H), 7.33-7.26 (m, 2H), 7.25-7.20 (m, 1H), 6.06 (s, 2H), 4.21 (s, 2H).

Step 2: To a solution of **3a-S1** (0.390 g, 1.89 mmol) in AcOH (50 mL) was added Ethyl-4-chloroacetoacet-ate (0.371 g, 2.27 mmol). After stirring for 10 h at 90 °C, the reaction mixture was cooled to room temperature and cautiously poured over ice water, obtaining a precipitate that was filtered. The precipitate was crystallized by AcOH to give **3a-S2** (0.430mg, 68%) as white solid. ^{1}H NMR (400 MHz, DMSO-d_6) δ 7.50-7.42 (m, 2H), 7.36-7.29 (m, 2H), 7.29-7.23 (m, 1H), 6.16 (s, 1H), 4.66 (s, 2H), 4.45 (s, 2H).

Step 3: A 10 mL round bottom flask was charged with **3a-S2** (0.100 g, 0.33 mmol) and

phenylmethanamine (1.5 mL). After stirring for 0.5h at 90 °C, the reaction mixture was cooled to room temperature and cautiously poured over ethyl acetate. Petroleum ether was added to the reaction mixture until a precipitate is formed. The precipitate was crystallized by EtOH to afford **D2-3a** (0.074 g, 60%) as white solid.^{1}H NMR (400 MHz, DMSO-d_6) δ 7.54-7.48 (m, 2H), 7.43 (dtd, *J* = 2.6, 4.4, 5.5, 7.8 Hz, 5H), 7.35-7.26 (m, 2H), 7.27-7.19 (m, 1H), 5.60 (s, 1H), 4.40 (s, 2H), 4.14 (s, 2H), 3.93 (s, 2H).

5-((benzylamino)methyl)-2-((4-ethylbenzyl)thio)-[1,2,4]triazolo[1,5-a]pyrimidin-7-ol (D2-3b). The title compound was synthesized using the same procedure as that used for D2-3a except that 1-(chloromethyl)-4-ethylbenzene was used instead of chloromethylbenzene. White solid; yield 28%.^{1}H NMR (400 MHz, DMSO-d_6) δ 7.51 (dq, *J* = 2.5, 4.5 Hz, 2H), 7.43 (q, *J* = 5.9 Hz, 3H), 7.33 (d, *J* = 8.0 Hz, 2H), 7.13 (d, *J* = 7.8 Hz, 2H), 5.61 (d, *J* = 1.6 Hz, 1H), 4.36 (s, 2H), 4.15 (s, 2H), 3.94 (s, 2H), 2.56 (q, *J* = 7.6 Hz, 2H), 1.14 (t, *J* = 7.6 Hz, 3H).

5-((benzylamino)methyl)-N-(4-ethylphenyl)-7-hydroxy-[1,2,4]triazolo[1,5-a]pyrimidine-2-carboxamide (D2-4a).

Step 1: To a 100 mL round bottom flask was added 5-amino-1H-1,2,4-triazole-3-carboxylic acid (0.256 g, 2 mmol), EDCI (0.575 g, 3 mmol), HOBt (0.405 g, 3 mmol), and DMF (8 mL). The reaction was left to stir at 0 °C for 1h. To the flask was then added 4-ethylaniline (0.3 ml, 2.4 mmol) dissolved in DMF (8 mL), followed by DIPEA (1 ml, 6 mmol), and the reaction was left to stir overnight. The next day, the mixture was diluted with H_2O (20 mL) and extracted with EA (3 × 10 mL), and the combined organic phases were washed with brine, dried over Na_2SO_4, filtered, and concentrated. Moreover, purifying with flash chromatography (10% MeOH and 1% TEA in DCM) gives **4a-S1** as a white solid (0.200 g, 43%).^{1}H NMR (400 MHz, DMSO-d_6) δ 12.68 (s, 1H), 9.85 (s, 1H), 7.84-7.54 (m, 2H), 7.35-7.07 (m, 2H), 6.14 (s, 2H), 2.56 (q, *J* = 7.6 Hz, 2H), 1.16 (t, *J* = 7.6 Hz, 3H).

Step 2 and Step 3 was same as the procedures of **D2-3a** except that **4a-S1** and **4a-S2** were used instead of **3a-S1** and **3a-S2**. **D2-4a** was obtained as white solid (72 mg, 60%). ^{1}H NMR (400 MHz, DMSO-d_6) δ 10.22 (s, 1H), 7.78-7.68 (m, 2H), 7.59-7.50 (m, 2H), 7.48-7.39 (m, 3H), 7.22-7.14 (m, 2H), 5.73 (s, 1H), 4.22 (s, 2H), 4.03 (s, 2H), 2.57 (t, *J* =

7.6 Hz, 2H), 1.18 (t, *J* = 7.6 Hz, 3H).

4. Synthesis of FDW028-PEG6-biotin.

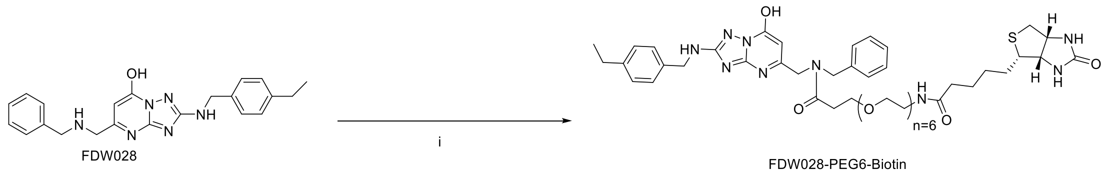

Scheme 4. Synthesis of FDW028-PEG6-biotin. (i) Biotin-PEG6-acid, EDCI, HOBT, DIPEA, DMF, r.t., 10 h.

Step 1: To a 10 mL round bottom flask was added Biotin-PEG6-acid (0.148g, 0.26 mmol), EDCI (0.064 mg, 0.38 mmol), HOBt (0.052g, 0.38mol), and DMF (7mL). The reaction was left to stir at 0 °C for 1h. To the flask was then added **FDW028** (0.100g, 0.26 mmol) dissolved in DMF (3 mL), followed by DIPEA (0.100 g, 0.78 mmol), and the reaction was left to stir overnight. The next day, the mixture was diluted with H_2O (10 mL) and extracted with EA (3 × 10 mL), and the combined organic phases were washed with brine, dried over Na_2SO_4, filtered, and concentrated. Then, purify with flash chromatography (10% MeOH in DCM) gives **FDW028-PEG6-biotin** as light yellow oil (0.020g, 8%). LCMS (ESI) m/z: calculated for $C_{47}H_{67}N_9O_{10}S$ $[M + H]^+$ 950.5 found 949.7, calculated for $C_{47}H_{67}N_9O_{10}S$ $[M+Na]^+$ 972.5 found 971.7, calculated for $C_{47}H_{67}N_9O_{10}S$ $[M-H]^-$ 948.5 found 947.7.

TABLES

Table S1 The primers utilized for qPCR assays.

Gene	Forward primer (5'→3')	Reverse primer (5'→3')
FUT1	GTGCCCGTATCCAGAGTGAT	AGGACCCAGGGGAGAGTAAA
FUT2	CTACCACCTGAACGACTGGATG	AGGGTGAACTCCTGGAGGATCT
FUT3	GCCGACCGCAAGGTGTAC	TGACTTAGGGTTGGACATGATATCC
FUT4	TCCTACGGAGAGGCTCAG	TCCTCGTAGTCCAACACG
FUT5	TATGGCAGTGGAACCTGTCA	CGTCCACAGCAGGATCAGTA
FUT6	CATTTCTGCTGCCTCAGG	GGGCAAGTCAGGCAACTC
FUT7	GAGAATCTCGGGTCTCTTGG	CAGACAAGGATGGTGATCGTG
FUT8	GACAGAACTGGTTCAGCGGAGA	GCAGTAGACCACATGATGGAGC
FUT9	CACCGAGACATCAGTTGGGATCT	CTTGGATATCTGAATCACGGCGG
FUT10	ATCTTGCCTGTGCGTCAC	ATGCGTAGGTGCTTCCTC
FUT11	CTCTTGGCTTTCTTGTCC	ATGACGGAGTGATTGTTC
POFUT1	CCAGCATCACAAGCCTCCTTT	CTCTGGCTCCATTGTTCTCTGT
POFUT2	TTCACGACCACTATGGAGG	CGGAGCCCAGCTTGACCTT
HSC70	GCTATGTCGCCTTTACGGACA	GCCAGCATCATTCACCACCATA
LAMP2A	GTTTCAGTGTCTGGAGCATTTC	GCACAAGGAAGTTGTCGTCA
GAPDH	TGCACCACCAACTGCTTAGC	GGCATGGACTGTGGTCATGAG

Table S2 LC-MS/MS analysis of the proteins pulled down by B7-H3 in SW480 cells.

Accession	Protein Name	# AAs	MW [kDa]	Area	emPAI	Score Sequest HT	Coverage Sequest HT
O75688	PPM1B	479	52.609	1.70E+07	0.532	17.65069282	9.603340292
Q5ZPR3	CD276	534	57.199	1.10E+07	0.274	11.84529543	5.243445693
Q16629	SRSF7	238	27.35	9.10E+06	0.155	3.829475522	3.781512605
P11166	SLC2A1	492	54.049	8.40E+06	0.145	0	2.032520325
P02647	APOA1	267	30.759	6.90E+06	0.389	8.031313896	4.119850187
E9PR30	FAU	98	10.898	5.80E+06	0.468	2.288972139	10.20408163
Q9BQA1	WDR77	342	36.701	5.50E+06	0.389	10.3725183	8.479532164
C9JC84	FGG	461	52.304	4.70E+06	0.083	1.717566609	1.952277657
A0A0U1RRH7	Histone H2A	170	18.541	4.60E+06	0.233	4.21283412	5.294117647
P61254	RPL26	145	17.248	4.50E+06	0.292	1.791257858	6.206896552
P04085	PDGFA	211	24.028	4.30E+06	0.233	1.734282494	3.791469194
P19474	TRIM21	475	54.135	4.30E+06	0.16	6.298930645	3.789473684
O14744	PRMT5	637	72.638	3.90E+06	0.129	6.516376853	2.982731554
P02649	APOE	317	36.132	3.70E+06	0.096	3.703071833	2.839116719
U3KQK0	HIST1H2BN	166	18.792	3.40E+06	0.425	1.781346083	14.45783133
Q9NZT1	CALML5	146	15.883	3.00E+06	0.292	1.781986237	5.479452055
P68371	TUBB4B	445	49.799	2.90E+06	0.35	8.636849165	9.213483146
P02545	LMNA	664	74.095	2.80E+06	0.053	4.37276423	1.355421687
Q5T749	KPRP	579	64.093	2.70E+06	0.105	4.417414427	1.554404145
Q9BRL6	SRSF8	282	32.268	2.50E+06	0.179	1.676411867	2.836879433
P07437	TUBB	444	49.639	2.40E+06	0.35	8.648949862	9.234234234
A0A2U3TZH3	EEF1A2	496	54.306	2.20E+06	0.292	5.615974665	5.241935484
P05783	KRT18	430	48.029	2.00E+06	0.359	4.020526528	6.279069767

Accession	Protein Name	# AAs	MW [kDa]	Area	emPAI	Score Sequest HT	Coverage Sequest HT
H3BSS5	KCTD5	246	27.109	2.00E+06	0.166	1.965274334	4.87804878
P62937	PPIA	165	18.001	2.00E+06	0.179	1.894652247	8.484848485
P26373	RPL13	211	24.247	1.70E+06	0.389	3.734298706	8.530805687
P07355	ANXA2	339	38.58	1.70E+06	0.101	2.231539488	2.654867257
Q15393	SF3B3	1217	135.492	1.60E+06	0.036	2.378240108	0.986031224
P11142	HSPA8	646	70.854	1.60E+06	0.061	4.249559402	2.012383901
B7Z4C8	RPL31	130	15.109	1.60E+06	0.334	0	6.923076923
O95881	TXNDC12	172	19.194	1.40E+06	0.194	0	5.23255814
P21333	FLNA	2647	280.564	1.40E+06	0.031	4.847812653	0.98224405
P09651	HNRNPA1	372	38.723	1.40E+06	0.122	1.812566996	2.688172043
E9PK25	CFL1	204	22.714	1.30E+06	0.179	0	5.392156863
P14618	PKM	531	57.9	1.20E+06	0.064	1.674449205	1.506591337
Q9Y3U8	RPL36	105	12.246	1.10E+06	0.468	0	9.523809524
P62258	YWHAE	255	29.155	9.70E+05	0.136	0	3.921568627
Q02878	RPL6	288	32.708	9.60E+05	0.129	1.602821469	3.125
P83731	RPL24	157	17.768	9.50E+05	0.259	0	5.732484076
P15924	DSP	2871	331.569	8.70E+05	0.011	1.699917436	0.313479624
P81605	DCD	110	11.277	8.50E+05	0.292	1.832010746	10.90909091
Q7RTV0	PHF5A	110	12.397	8.40E+05	0.334	2.178889513	11.81818182
P14923	JUP	745	81.693	7.50E+05	0.054	2.451729059	1.879194631
P54105	CLNS1A	237	26.199	7.20E+05	0.233	1.891435623	5.485232068
P62269	RPS18	152	17.708	6.10E+05	0.212	1.611881495	7.236842105
A0A3B3ITT5	RPL29	167	18.5	5.40E+05	0.389	0	4.790419162
Q02543	RPL18A	176	20.749	4.90E+05	0.179	1.968677998	7.386363636
Q14240	EIF4A2	407	46.373	4.60E+05	0.101	0	2.457002457

Accession	Protein Name	# AAs	MW [kDa]	Area	emPAI	Score Sequest HT	Coverage Sequest HT
P07900	HSP90AA1	732	84.607		0.056	2.138608217	2.049180328
O75367	H2AFY	372	39.592		0.129	0	7.795698925

Table S3 Compound D2 and its derivatives.

Compound ID	Structure
D2-1a	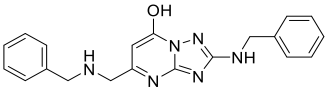
D2-1b	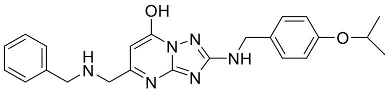
D2-1c (FDW028)	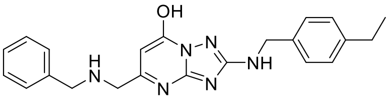
D2-1d	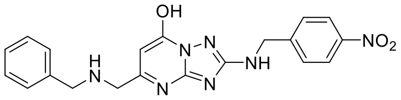
D2-1e	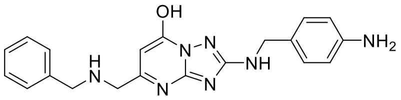
D2-1f	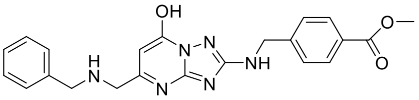
D2-1g	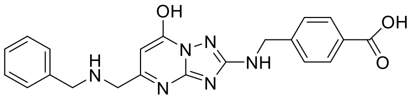
D2-2a	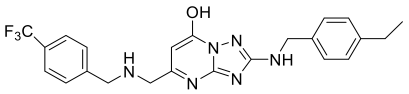
D2-2b	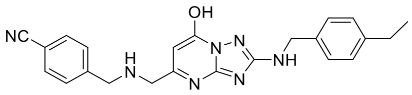
D2-2c	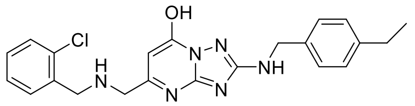
D2-2d	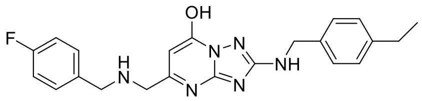
D2-2e	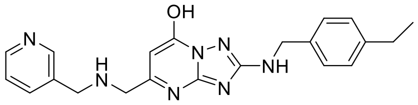
D2-2f	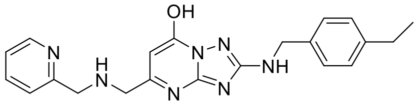
D2-3a	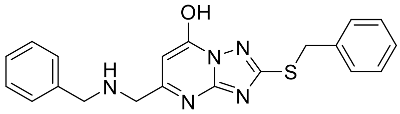

Compound ID	Structure
D2-3b	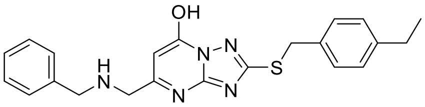
D2-4a	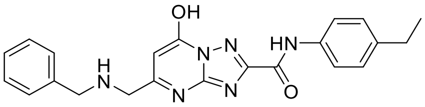

Figures

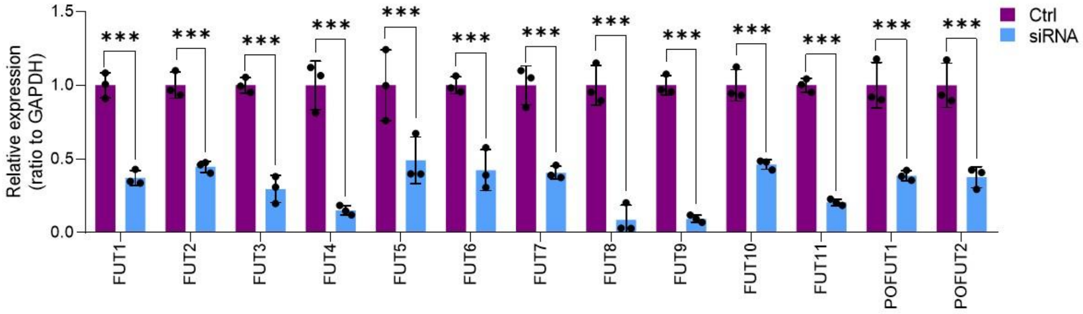

Figure S1 Validation of siRNAs of FUTs.

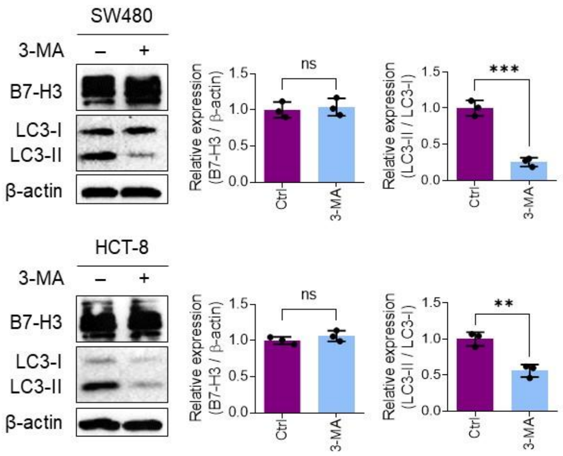

Figure S2 Immunoblots of B7-H3 in SW480 and HCT-8 cells with the treatment of 3-MA.

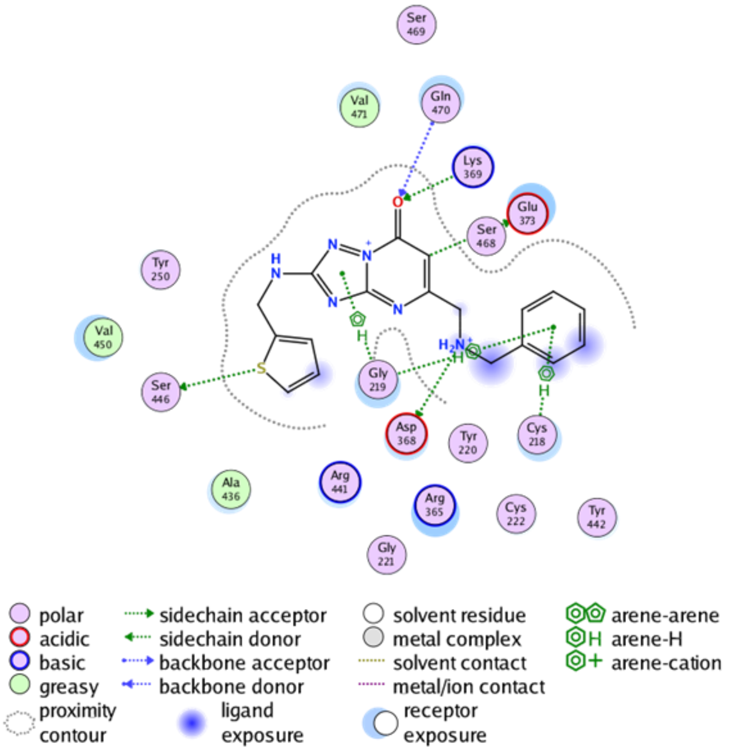

Figure S3 The 2D docking of compound D2 with FUT8 protein.

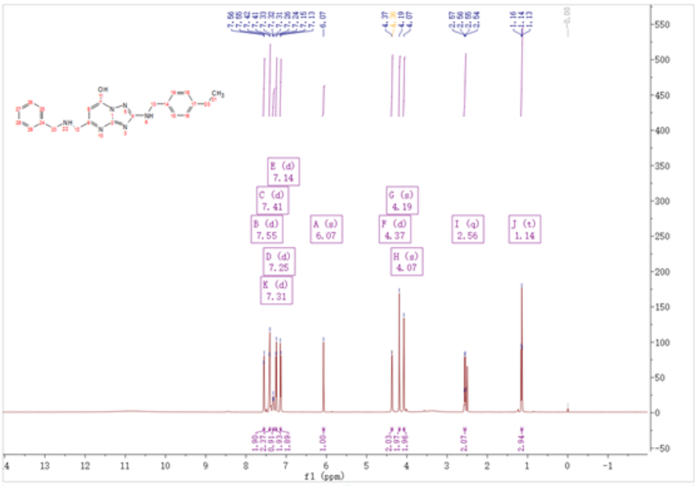

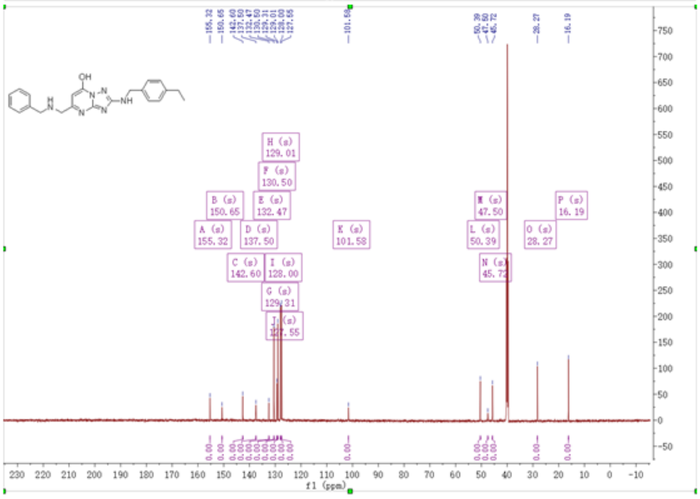

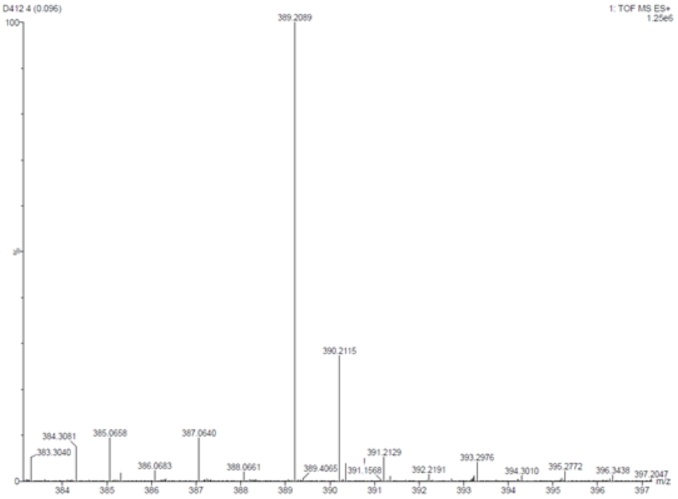

Figure S4 ^{1}H NMR spectrum, ^{13}C NMR spectrum, and HRMS of FDW028.

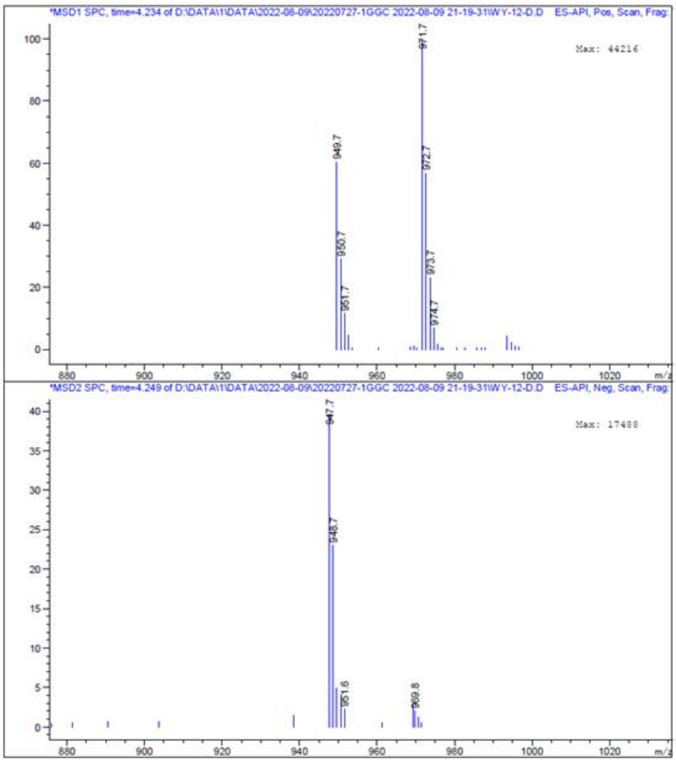

Figure S5 The MS spectrum of FDW028-PEG6-biotin.

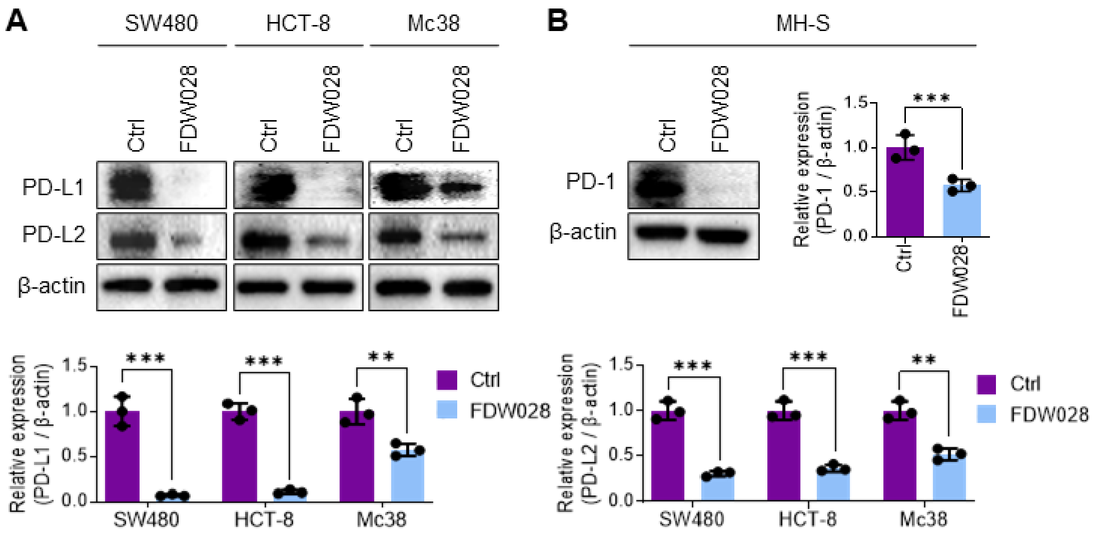

Figure S6 The effect of FDW028 on the expression of PD-L1/2 and PD-1**. A** Immunoblots of PD-L1 and PD-L2 in SW480, HCT-8 and Mc38 cells treated with FDW028 at 50 μM for 72 h. **B** Immunoblots of PD-1 in MH-S cells treated with FDW028 at 50 μM for 72 h. All experiments were performed in technical triplicates and are displayed as mean ± s.d.; **, *P*<0.01; ***, *P*<0.001.
